# The KDM4B–CCAR1–MED1 axis is a critical regulator of osteoclast differentiation and bone homeostasis

**DOI:** 10.1038/s41413-021-00145-1

**Published:** 2021-05-25

**Authors:** Sun-Ju Yi, You-Jee Jang, Hye-Jung Kim, Kyubin Lee, Hyerim Lee, Yeojin Kim, Junil Kim, Seon Young Hwang, Jin Sook Song, Hitoshi Okada, Jae-Il Park, Kyuho Kang, Kyunghwan Kim

**Affiliations:** 1 Department of Biological Sciences and Biotechnology, Chungbuk National University, Cheongju, Chungbuk Republic of Korea; 2 Korea Basic Science Institute, Gwangju Center at Chonnam National University, Gwangju, Republic of Korea; 3 New Drug Development Center, KBIO Osong Medical Innovation Foundation, Cheongju, Chungbuk Republic of Korea; 4 Data Convergence Drug Research Center, Therapeutics & Biotechnology Division, Korea Research Institute of Chemical Technology, Daejeon, Republic of Korea; 5 Department of Biochemistry, Kindai University Faculty of Medicine, Osakasayama, Osaka Japan

**Keywords:** Bone, Pathogenesis

## Abstract

Bone undergoes a constant and continuous remodeling process that is tightly regulated by the coordinated and sequential actions of bone-resorbing osteoclasts and bone-forming osteoblasts. Recent studies have shown that histone demethylases are implicated in osteoblastogenesis; however, little is known about the role of histone demethylases in osteoclast formation. Here, we identified KDM4B as an epigenetic regulator of osteoclast differentiation. Knockdown of KDM4B significantly blocked the formation of tartrate-resistant acid phosphatase-positive multinucleated cells. Mice with myeloid-specific conditional knockout of KDM4B showed an osteopetrotic phenotype due to osteoclast deficiency. Biochemical analysis revealed that KDM4B physically and functionally associates with CCAR1 and MED1 in a complex. Using genome-wide chromatin immunoprecipitation (ChIP)-sequencing, we revealed that the KDM4B–CCAR1–MED1 complex is localized to the promoters of several osteoclast-related genes upon receptor activator of NF-κB ligand stimulation. We demonstrated that the KDM4B–CCAR1–MED1 signaling axis induces changes in chromatin structure (euchromatinization) near the promoters of osteoclast-related genes through H3K9 demethylation, leading to NF-κB p65 recruitment via a direct interaction between KDM4B and p65. Finally, small molecule inhibition of KDM4B activity impeded bone loss in an ovariectomized mouse model. Taken together, our findings establish KDM4B as a critical regulator of osteoclastogenesis, providing a potential therapeutic target for osteoporosis.

## Introduction

Bone provides structural support, protects vital organs and tissues, and stores minerals such as calcium and phosphates. Bone is a dynamic organ that undergoes a remodeling process regulated by the coordinated and sequential actions of bone-resorbing osteoclasts and bone-forming osteoblasts.^1,2^ Osteoclasts, giant multinucleated cells, differentiate from mononuclear hematopoietic precursors upon stimulation by receptor activator of NF-κB ligand (RANKL).^3^ The binding of RANKL to its receptor RANK on osteoclast precursor (OCP) cell membranes results in the recruitment and stimulation of tumor necrosis factor receptor-associated factor 6, followed by the activation of multiple signaling pathways, including the NF-κB, mitogen-activated protein kinase, and AKT pathways. The coordinated activation of transcription factors such as NF-κB, c-Fos, c-Jun, and microphthalmia-associated transcription factor induces the expression of nuclear factor of activated T cells c1 (NFATc1), a master transcription factor for osteoclast differentiation. NFATc1 is essential for the expression of osteoclastogenic genes encoding tartrate-resistant acid phosphatase (TRAP), cathepsin K, matrix metalloproteinase 9, and beta 3-integrins.^4–6^ Aberrant regulation of osteoclast formation can contribute to the pathogenesis of bone diseases such as osteoporosis, osteopetrosis, and Paget’s disease of bone.^7^

The differentiation and activity of osteoclasts require a multistep differentiation process accompanied by the expression of osteoclastogenic genes, which are regulated by transcription factors, coactivators, and corepressors. An increasing number of studies have defined epigenetic changes as determinants of gene expression in bone remodeling.^8–12^ Histone-modifying enzymes, in particular, have been reported to play a pivotal role in the expression of osteoclastogenesis-related genes.^13–18^ Although progress has been made in exploring the changes in histone modifications during osteoclastogenesis, the relationship between histone-modifying enzymes and osteoclast differentiation remains incompletely defined.

The enzyme Jumonji C (JmjC)-domain containing demethylase (KDM/JMJD) catalyzes the demethylation of specific mono-, di-, and trimethylated lysine residues on histones, regulating chromatin structure and gene expression.^19,20^ The KDM4 (JMJD2) subfamily of JMJD proteins has been reported to be involved in diverse biological processes, including development, spermatogenesis, differentiation, hematopoietic stem cell maintenance, and tumorigenesis.^21–27^ The KDM4A-C proteins, which share more than 50% sequence identity, contain a JmjN domain, a JmjC domain, two plant homeodomains, and double Tudor domains, while KDM4D and KDM4E are considerably shorter proteins lacking the C-terminal region, which includes the plant homeodomains and Tudor domains.^28^ KDM4A-C is overexpressed in many cancers, including breast, colorectal, lung, prostate, and gastric cancers, and is implicated in the establishment and progression of these cancers.^29^ Notably, several studies have revealed that KDM4B is a conserved epigenetic regulator linked to diverse developmental processes, such as mammary gland development,^30^ neural development,^31^ inner ear development,^32^ self-renewal and differentiation of stem cells,^23,33^ chondrogenic differentiation,^34^ myogenesis,^35^ and adipogenesis.^36,37^ These observations suggest the possibility that KDM4B may be involved in osteoclast differentiation.

In this study, we identified KDM4B as an epigenetic regulator of osteoclast differentiation. We found that RANKL signaling induced the expression and stabilization of KDM4B. Ex vivo deletion of *Kdm4b* using shRNA and studies in myeloid-specific *Kdm4b*-deficient mice clearly demonstrated that KDM4B is crucial for osteoclast formation and bone homeostasis. These biochemical and genome-wide studies showed that KDM4B–CCAR1–MED1 signaling induces a change from a heterochromatic to a euchromatic environment, subsequently facilitating recruitment of NF-κB p65 to the promoters of osteoclastogenic genes and their subsequent expression.

## Results

### *Kdm4b* is critical for RANKL-induced osteoclast formation

**Fig. 1:**
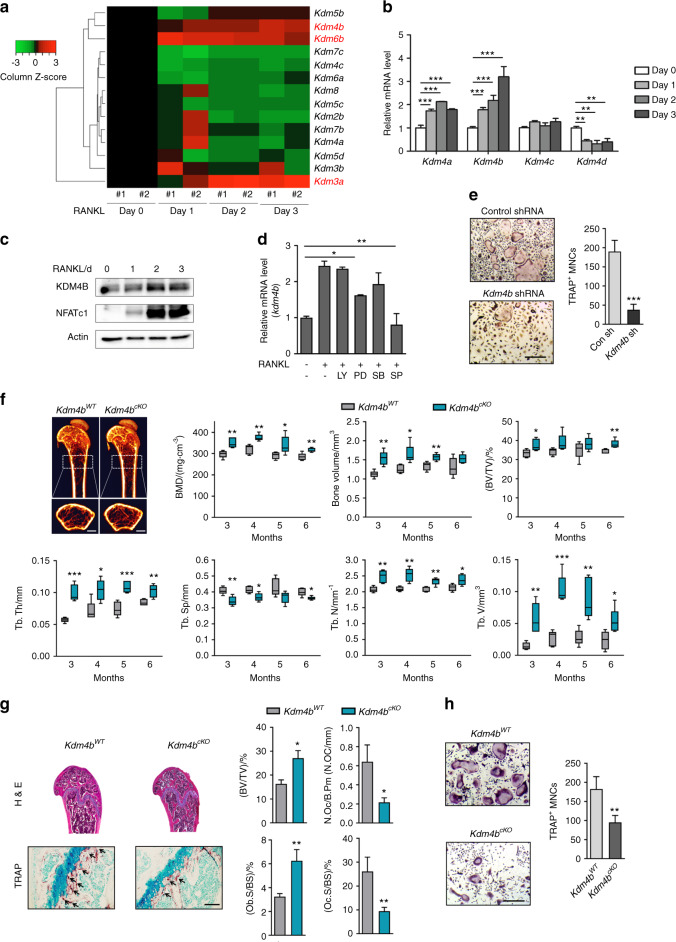
Myeloid-specific loss of KDM4B increases bone mass and reduces osteoclast activity in vivo. **a** Heatmap showing mRNA expression profiles of histone demethylases during osteoclastogenesis. **b** Relative mRNA levels of *Kdm4a*–*Kdm4d* during osteoclast differentiation. The mRNA levels of each reaction were normalized to the mRNA level of the β–actin control. **c** Immunoblot analysis of KDM4B expression levels during osteoclastogenesis. **d** Effects of protein kinase inhibitors on the mRNA expression of *Kdm4b* (LY, LY294002; PD, PD98059; SB, SB203580; SP, SP600125). **e** TRAP staining of BMMs expressing either control shRNA or *Kdm4b* shRNA. Scale bar, 75 μm. **f** Micro-CT analysis of the femurs of 3-month-old male *Kdm4b*^WT^ and *Kdm4b*^cKO^ mice (*n* = 5). Representative micro-CT image of the proximal femur (top, longitudinal view; bottom, axial view). The boxes enclose the 25th to 75th percentile values. The whiskers extend to the maximum and minimum values. The central horizontal bar indicates the median. Scale bars, 1 mm. **g** Histological analysis of femur sections from 3-month-old male *Kdm4b*^WT^ and *Kdm4b*^cKO^ mice (*n* = 3). The sections were stained for H&E and TRAP. The arrows indicate osteoclasts. Scale bar, 100 μm. **h** TRAP staining of BMMs from Kdm4b^WT^ and Kdm4b^cKO^ mice. Scale bar, 75 μm. The data are presented as the mean ± SD values of three independent experiments [two-way ANOVA in (**b**), one-way ANOVA in (**d**), two-tailed *t*-test in (**e**, **h**)]. In (**f**, **g**), the data are presented as the mean ± SEM values. *P < 0.05; **P < 0.01; ***P < 0.001. See also Supplementary Fig. 1

To study the possible epigenetic role(s) of histone demethylases during osteoclastogenesis, we first examined the expression levels of histone demethylases using MouseRef-8 expression microarrays. We found that three genes, *Kdm3a*, *Kdm4b*, and *Kdm6b*, were gradually upregulated after RANKL treatment (Fig. 1a). As *Kdm4b* is strongly expressed during osteoclastogenesis and is implicated in cell differentiation,^23,33,35–37^ studying its possible effects on osteoclast formation was a logical extension of our study. Through RT-PCR and western blot analysis, we confirmed that *Kdm4b* expression is increased during osteoclast differentiation (Fig. 1b, c). Intriguingly, our studies revealed that JNK signaling was associated with *Kdm4b* expression (Fig. 1d). We next investigated whether *Kdm4b* expression affects osteoclast formation. Depletion of *Kdm4b* significantly inhibited RANKL-induced osteoclast differentiation (Fig. 1e and Supplementary Fig. 1a). Furthermore, the finding that *Kdm4b* knockdown did not affect the proliferation of preosteoclasts suggested that *Kdm4b* selectively suppresses the differentiation but not the proliferation of OCPs (Supplementary Fig. 1b). Next, we determined whether *Kdm4b* can regulate the expression of NFATc1 and its target genes. Knockdown of *Kdm4b* significantly disrupted the expression of NFATc1 and three of its target genes after RANKL treatment (Supplementary Fig. 1c). These ex vivo results suggested that *Kdm4b* is transcriptionally activated via the RANKL-JNK signaling pathway and subsequently activates RANKL-induced osteoclast formation.

### Loss of myeloid-specific *Kdm4b* enhances bone mineral density in vivo

The finding that knockdown of *Kdm4b* in OCPs suppresses RANKL-mediated osteoclast formation prompted us to explore the biological significance of *Kdm4b* expression in vivo. We generated myeloid-specific conditional knockout mice by crossing *Kdm4b*^f/f^ mice with Lyz2-Cre transgenic (hereafter *Kdm4b*^cKO^) mice. Bone architecture from 3 to 6 months of age was observed by micro-CT analysis. In *Kdm4b*^cKO^ mice, bone mass was greatly enhanced compared to that in littermate control (*Kdm4b*^f/f^, hereafter *Kdm4b*^WT^) mice (Fig. 1f). Bone histomorphometric analysis revealed that trabecular osteoclast numbers and trabecular surface area, as well as bone resorption activity, were decreased in *Kdm4b*^cKO^ mice. Unexpectedly, Ob.S/BC, one of the bone formation-related parameters, was increased in cKO mice (Fig. 1g). These results suggest that *Kdm4b* loss resulted in a high bone mass phenotype associated with both decreased osteoclast and increased osteoblast activity. We next performed ex vivo osteoclast differentiation assays using OCPs from *Kdm4b*^cKO^ and *Kdm4b*^WT^ mice. As expected, *Kdm4b* deficiency significantly diminished osteoclast formation (Fig. 1h and Supplementary Fig. 1d). Consistent with this finding, the mRNA expression level of *Nfatc1* was also reduced in *Kdm4b*^cKO^ OCPs (Supplementary Fig. 1e). These results suggest that *Kdm4b* is necessary for osteoclast differentiation and bone resorption.

### CCAR1 is a KDM4B-interacting protein and is involved in osteoclast formation

**Fig. 2:**
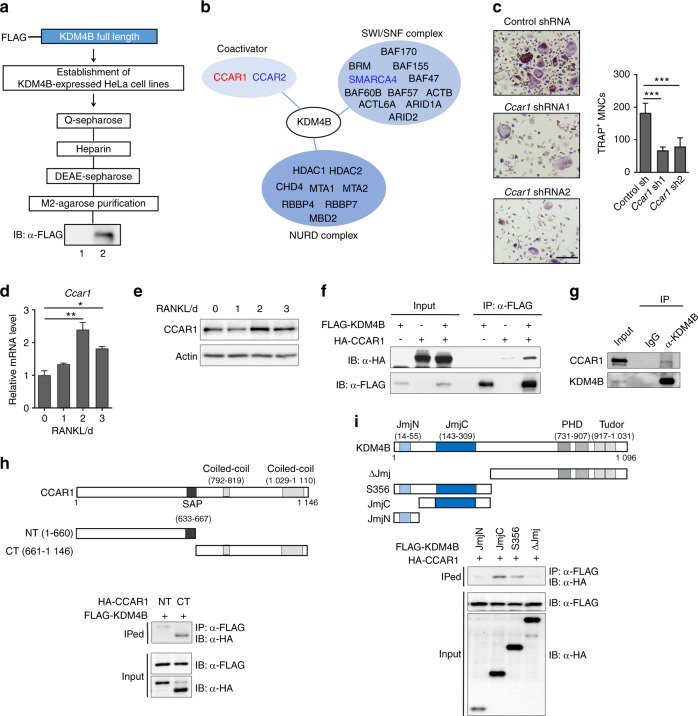
KDM4B associates with CCAR1. **a** Schematic diagram of the method used to purify the KDM4B-interacting complex from FLAG-KDM4B-expressing cells. Lane 1, control preparation from the nuclear extract of normal HeLa cells; lane 2, KDM4B-interacting complex from the nuclear extract of KDM4B-expressing HeLa cells. **b** KDM4B-interacting complexes. **c** TRAP staining of BMMs expressing either control shRNA or *Ccar1*1 shRNA1. Scale bar, 75 μm. **d**, **e** CCAR1 expression during osteoclast differentiation. qRT-PCR (**d**) and immunoblot (**e**) results. **f** Coimmunoprecipitation of FLAG-KDM4B and HA-CCAR1. **g** Endogenous interaction of KDM4B and CCAR1 in BMMs. **h**, **i** Mapping the binding domain of KDM4B and CCAR1. JmjN N-terminal jumonji domain, JmjC jumonji C-terminal domain, PHD plant homeodomain finger domain, Tudor Tudor domain. SAP, SAF-A/B, Acinus, and PIAS DNA/RNA binding motif. The data are presented as the mean ± SD values of three independent experiments [one-way ANOVA in (**c**, **d**)]. *P < 0.05; **P < 0.01; ***P < 0.001. See also Supplementary Fig. 2

Recent studies demonstrated that KDM4B functions as a transcriptional coactivator by regulating H3K9 methylation.^23,33,38,39^ To further understand the biological role of KDM4B in euchromatinization, we generated a HeLa cell line stably expressing KDM4B fused to FLAG and HA epitope tags. Nuclear extracts were initially fractionated by Q-Sepharose chromatography; fractions containing ectopic KDM4B were combined and subjected to sequential column chromatography in heparin and DEAE Sepharose and M2 agarose columns (Fig. 2a). Proteins copurified with KDM4B were analyzed by tandem mass spectrometry. KDM4B physically associates with the SWI/SNF complex, nucleosome remodeling deacetylase (NURD) complex, and additional coactivators (Fig. 2b).^30,40–43^ As we sought to find interacting partners involved in euchromatinization, the NURD corepressor complex was excluded from our studies. To determine which coactivators functionally associate with KDM4B during osteoclast formation, we used shRNA targeting CCAR1, CCAR2, or SMARCA4 (a subunit of the SWI/SNF complex) to deplete these genes. The targeted gene depletion studies revealed that CCAR1 was required for osteoclast formation, whereas CCAR2 and the rest of the SWI/SNF complex had either no effect or less effect than CCAR1 on osteoclastogenesis (Fig. 2c and Supplementary Fig. 2a, b). Moreover, concomitant with *Kdm4b*, *Ccar1* was transcriptionally increased upon RANKL treatment, and CCAR1 regulated the mRNA expression of *Nfatc1* and its target genes (Fig. 2d, e and Supplementary Fig. 2c). These results strongly suggested that KDM4B functionally interacts with CCAR1.

Next, we sought to determine whether KDM4B physically interacts with CCAR1. We transfected cells with FLAG-KDM4B and HA-CCAR1 and performed coimmunoprecipitation (co-IP) assays using an anti-FLAG antibody. We observed a direct interaction between KDM4B and CCAR1 (Fig. 2f). These data were further supported by the interaction between endogenous KDM4B and CCAR1 in bone marrow-derived macrophages (BMMs) (Fig. 2g). To more precisely determine the nature of the association between KDM4B and CCAR1, we performed additional co-IP experiments in cells expressing a series of KDM4B and CCAR1 truncation mutants. In mapping the KDM4B-interacting region of CCAR1, the C-terminal region of CCAR1 (residues 661–1146) retained binding affinity for KDM4B, but no apparent interaction was detected in the N-terminal region of CCAR1 (Fig. 2h). In similar binding experiments mapping the CCAR1-interacting region of KDM4B, both S356 and the JmjC domain were determined to be required for its binding to CCAR1, whereas the JmjN domain was not required (Fig. 2i), indicating that the JmjC domain plays a role in KDM4B binding to CCAR1.

### KDM4B and CCAR1 are colocalized to and act at osteoclast-related genes

**Fig. 3:**
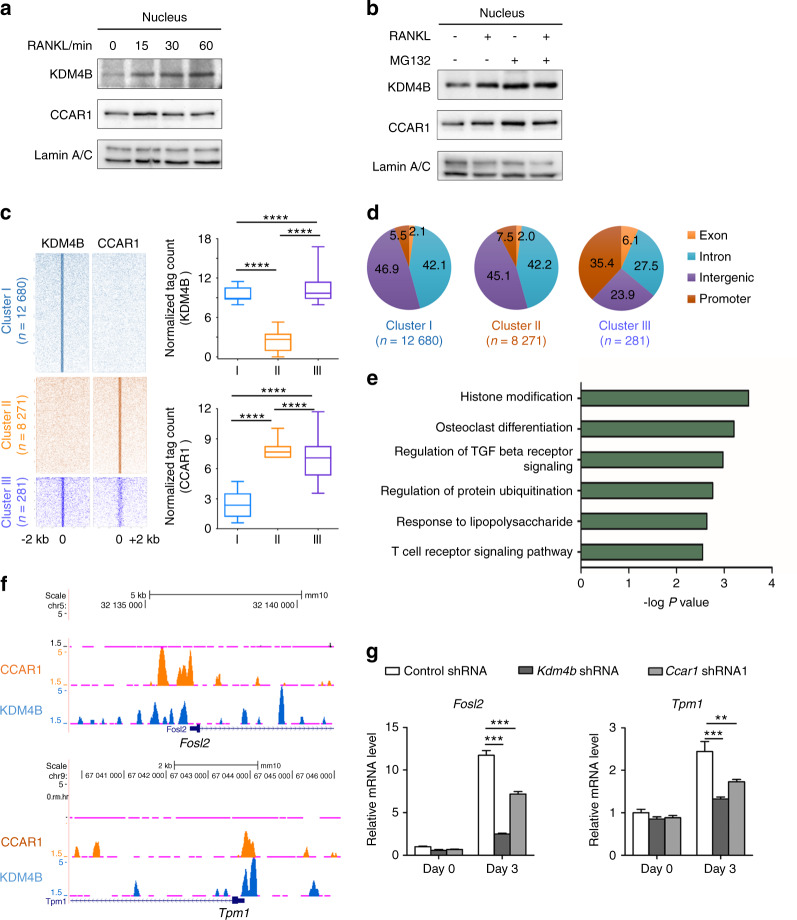
KDM4B and CCAR1 are colocalized near TSSs and enhance the gene expression of a set of osteoclast-related genes. **a** Immunoblots of nuclear fractions of BMMs after RANKL treatment. **b** KDM4B stabilization by RANKL or MG132. **c** Heatmap of normalized tag densities on KDM4B and CCAR1 peaks (left panels). Quantifications of tag counts are shown in the box plots (right panels). The boxes enclose the 25th to 75th percentile values. The whiskers extend to the 10th and 90th percentiles. The central horizontal bar indicates the median. ****P < 0.000 1 (Kolmogorov–Smirnov test). **d** Genomic locations of binding sites within each cluster are expressed as percentages of the total binding sites. **e** Gene Ontology (GO) enrichment analysis of Cluster III. **f** Representative UCSC Genome Browser tracks showing co-occupancy of KDM4B and CCAR1 at *Fosl2* and *Tpm1*. **g** mRNA expression levels of *Fosl2* and *Tpm1* in cells expressing control shRNA, *Kdm4b* shRNA, or *Ccar1* shRNA1. The data are presented as the mean ± SD of three independent experiments (two-way ANOVA). **P < 0.01; ***P < 0.001. See also Supplementary Fig. 3

To examine the potential molecular mechanisms underlying the chromatin-related function of KDM4B and CCAR1 during RANKL-mediated osteoclastogenesis, we performed chromatin immunoprecipitation (ChIP)-seq analysis with KDM4B and CCAR1 after RANKL treatment. Given that KDM4B and CCAR1 are localized in both the cytosol and in the nucleus^41,44^ and that RANKL-RANK signaling is rapidly transmitted to the nucleus, we speculated that RANKL-RANK signaling can trigger nuclear accumulation of KDM4B and CCAR1. We first verified the subcellular distribution of KDM4B and CCAR1. A time-course analysis showed that most CCAR1 was localized in the nucleus in the absence of RANKL treatment and that RANKL signaling did not change its subcellular localization (Fig. 3a and Supplementary Fig. 3a). Interestingly, we observed that KDM4B rapidly accumulated in the nucleus within 15 min after RANKL treatment, with no change in its cytoplasmic localization. These results suggested that RANKL signaling stabilizes nuclear KDM4B instead of inducing nuclear translocation of cytoplasmic KDM4B. We then sought to determine whether the proteasome inhibitor MG132 affects KDM4B stability. Our western blot results clearly showed that KDM4B degradation was blocked by treatment with MG132 (Fig. 3b), indicating that KDM4B expression is regulated by proteasomal degradation.

Based on the above results, we performed ChIP-seq analysis with KDM4B and CCAR1 following 30 min of treatment with RANKL. The heatmap of the ChIP-seq profiles shows three distinct groups based on the overlap of KDM4B and CCAR1 peaks (Fig. 3c, left): KDM4B specific (Cluster I, *n* = 12 680), CCAR1 specific (Cluster II, *n* = 8 271), or both (Cluster III, *n* = 281). Profiling the normalized tag counts in each group demonstrated that Cluster III was distinct from the other two clusters (Fig. 3c, right). Interestingly, genome-wide location analysis showed that 35.4% of the peaks identified in Cluster III mapped to promoters, whereas only a small portion of the peaks in the two other clusters (7.5% in Cluster II and 5.5% in Cluster I) mapped to promoters (Fig. 3d). These results indicate that colocalization of KDM4B and CCAR1 might be functionally related to target gene expression. Gene Ontology (GO) analysis revealed that Cluster III was mainly enriched in genes associated with histone modification and osteoclast differentiation (Fig. 3e). Several canonical osteoclastogenic genes, such as *Fosl2*, *Akt1*, *Gab2*, and *Tpm1*, were identified in Cluster III. Notably, KDM4B and CCAR1 were colocalized near the transcription start sites (TSSs) of *Fosl2*, *Akt1*, *Gab2*, and *Tpm1* (Fig. 3f and Supplementary Fig. 3b) and required for concomitant activation of their mRNA expression during osteoclastogenesis (Fig. 3g), strongly suggesting that KDM4B and CCAR1 modulate DNA transcription.

### The KDM4B–CCAR1–MED1 axis is essential for osteoclastogenesis

**Fig. 4:**
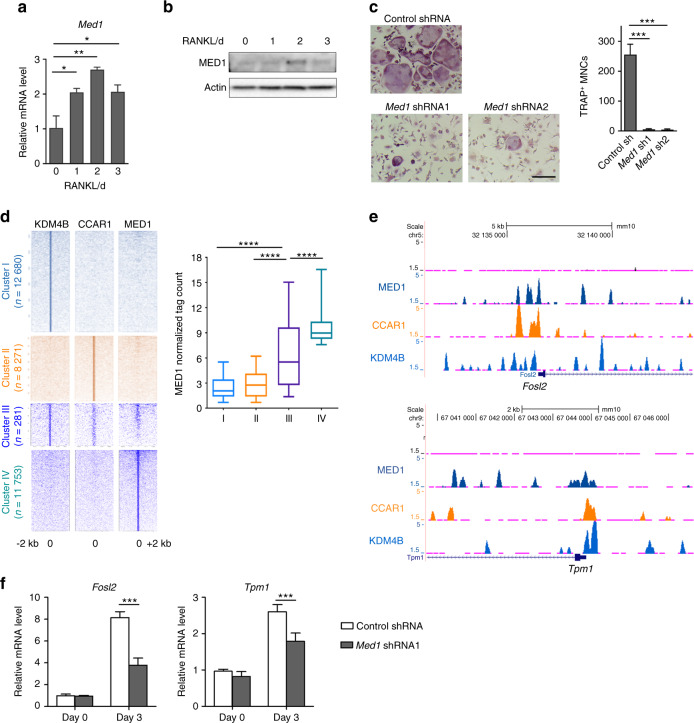
The KDM4B–CCAR1–MED1 axis is required for osteoclastogenesis. **a**, **b** MED1 expression during osteoclast differentiation. qRT-PCR (**a**) and immunoblot (**b**) results. **c** TRAP staining of BMMs expressing either control shRNA or *Med1* shRNA. Scale bar, 75 μm. **d** Heatmap of normalized tag densities on KDM4B, CCAR1, and MED1 peaks (2 kb up/downstream of the peak centers) (left panels). Quantifications of MED1 tag counts are shown in the box plots (right panel). The boxes enclose the 25th to 75th percentile values. The whiskers extend to the 10th and 90th percentiles. The central horizontal bar indicates the median. ****P < 0.000 1 (Kolmogorov–Smirnov test). **e** Representative UCSC Genome Browser tracks showing co-occupancy of KDM4B, CCAR1, and MED1 at *Fosl2* and *Tpm1*. **f** mRNA expression levels of *Fosl2* and *Tpm1* in cells expressing control shRNA or *Med1* shRNA. The data are presented as the mean ± SD values of three independent experiments [one-way ANOVA in (**a**, **c**)]. *P < 0.05; **P < 0.01; ***P < 0.001. See also Supplementary Fig. 4

It was previously reported that CCAR1 functions as a coactivator, facilitating MED1 and RNA polymerase II recruitment to promoters.^41,43,45^ To examine the functional role of MED1 in osteoclastogenesis, we first determined the expression level of MED1 upon RANKL treatment. The expression of MED1 was increased at the mRNA and protein levels (Fig. 4a, b). Depletion of MED1 significantly inhibited RANKL-induced osteoclast formation as well as the expression of osteoclast-related genes (Fig. 4c and Supplementary Fig. 4a, b), suggesting that MED1 might be functionally associated with the KDM4B–CCAR1 complex. Therefore, we sought to determine the genomic binding sites of MED1 during osteoclastogenesis to directly investigate the cooperative role of MED1 in transcriptional regulation. Integrated ChIP-seq analysis showed that MED1 co-occupies the same gene promoters in Cluster III, as shown in Fig. 3c, that are occupied by KDM4B and CCAR1 (Fig. 4d). Interestingly, pairwise enrichments of the three proteins were rarely detected. These results indicate that signaling via the KDM4B–CCAR1–MED1 axis may have a unique role in osteoclastogenesis. Consistent with this finding, MED1 co-occupied genomic regions near the TSSs of *Fosl2*, *Akt1*, *Gab2*, and *Tpm1* with KDM4B and CCAR1 (Fig. 4e and Supplementary Fig. 4c). Knockdown of MED1 significantly impaired *Fosl2* and *Tpm1* expression (Fig. 4f). These findings further indicate that KDM4B–CCAR1–MED1 is an essential axis of osteoclast differentiation.

**Fig. 5:**
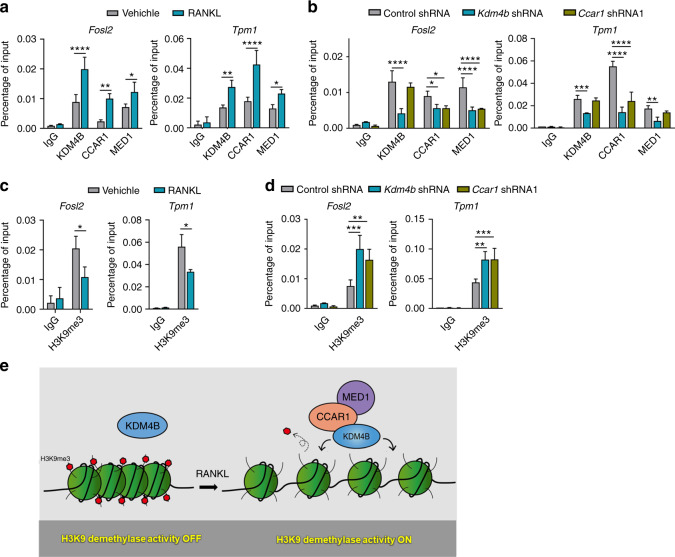
KDM4B-dependent recruitment of CCAR1 and MED1. **a** ChIP-qPCR of KDM4B, CCAR1, and MED1 enrichment upon RANKL treatment (100 ng·mL^−1^, 30 min) at the *Fosl2* (−1.1 kb) and *Tpm1* (−0.6 kb) *promoters*. **b** ChIP assays of KDM4B, CCAR1, and MED1 localization at target genes in cells depleted of KDM4B or CCAR1 upon RANKL signaling (100 ng·mL^−1^, 30 min). **c** ChIP-qPCR of H3K9me3 in the *Fosl2* and *Tpm1* promoters in the cells evaluated in (**a**). **d** ChIP assays of H3K9me3 status in the cells evaluated in (**b**) upon RANKL signaling. **e** Schematic representation of the cooperative function of KDM4B, CCAR1, and MED1 in regulating H3K9 demethylation. The data are presented as the mean ± SD values of three independent experiments [two-way ANOVA in (**a**–**d**)]. **P < 0.01; ***P < 0.001. See also Supplementary Fig. 5

To determine how KDM4B, CCAR1, and MED1 are recruited to target genes upon RANKL stimulation, we investigated their localization by ChIP. Crosslinked chromatin was isolated from control cells and cells depleted of KDM4B or CCAR1 (Supplementary Fig. 5a), and the precipitated DNA was amplified by qPCR using primers specific for the TSSs of *Fosl2* and *Tpm1*. In agreement with the ChIP-seq data, we observed that KDM4B, CCAR1, and MED1 were highly enriched around these TSSs upon RANKL treatment (Fig. 5a). Depletion of KDM4B reduced the levels of CCAR1 and MED1 at the TSSs (Fig. 5b). CCAR1 depletion diminished MED1 localization but did not lead to a substantial reduction in KDM4B occupancy. These results suggest that KDM4B initiates the recruitment and function of CCAR1 and MED1.

Since KDM4B demethylates H3K9me2/3, we determined the global levels of these two modifications by western blotting. Consistent with previous reports,^46,47^ loss of KDM4B did not affect the global H3K9 methylation status (Supplementary Fig. 5b). We next examined H3K9 trimethylation specifically at the *Fosl2* and *Tpm1* loci. Localization of KDM4B upon RANKL treatment was accompanied by a marked reduction in H3K9me3 at target genes (Fig. 5c). Depletion of KDM4B increased the levels of H3K9me3 (Fig. 5d), indicating that KDM4B controls gene expression through local rather than global H3K9me3 shifts in chromatin modification. The observed colocalization between KDM4B and CCAR1 and their function in mediating transcriptional activation raised the possibility that CCAR1 also plays a role in regulating the demethylase activity of KDM4B. As expected, CCAR1 depletion had no detectable effect on the global H3K9 methylation status (Supplementary Fig. 5b). In contrast, the local H3K9me3 level was sharply increased in response to CCAR1 knockdown (Fig. 5d). These results strongly indicate that KDM4B and CCAR1 cooperate to upregulate target genes by demethylating H3K9me3 (Fig. 5e).

### The KDM4B–CCAR1–MED1 signaling axis is required for p65-mediated osteoclastogenesis

**Fig. 6:**
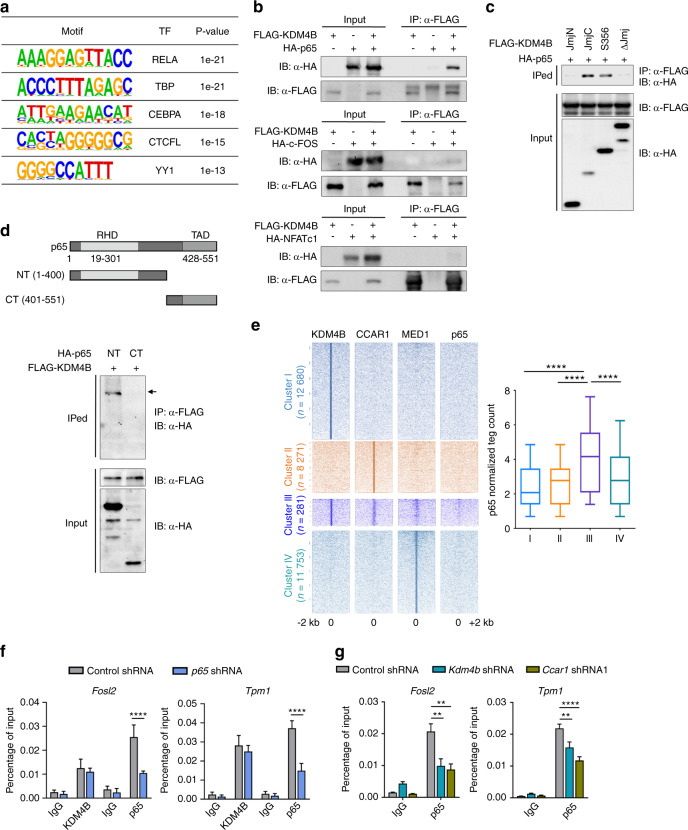
Association of p65 and KDM4B is critical for osteoclastogenesis. **a** The most significantly enriched transcription factor (TF) motifs at KDM4B binding regions, as identified by de novo motif analysis using HOMER. **b** KDM4B interaction with p65. **c**, **d** Mapping the binding domain of KDM4B and p65. RHD Rel homology domain, TAD transactivation domain. **e** Heatmap of normalized tag densities on KDM4B, CCAR1, MED1, and p65 peaks (left panels). Quantifications of p65 tag counts are shown in the box plots (right panel). The boxes enclose the 25th to 75th percentile values. The whiskers extend to the 10th and 90th percentiles. The central horizontal bar indicates the median. ****P < 0.000 1 (Kolmogorov–Smirnov test). **f** ChIP assay of KDM4B occupancy at the *Fosl2* or *Tpm1* promoter in p65-depleted cells. **g** ChIP assay of p65 occupancy at the *Fosl2* or *Tpm1* promoter in KDM4B- or CCAR1-depleted cells. The data are presented as the mean ± SD values of three independent experiments [two-way ANOVA in (**f**, **g**)]. **P < 0.01; ***P < 0.001. See also Supplementary Fig. 6

Given that KDM4B interacts with several transcription factors, including androgen receptors, estrogen receptor, and c-Jun,^26,30,38^ it was reasonable to examine transcription factors correlated with KDM4B during osteoclastogenesis. To this end, de novo motif analysis of KDM4B ChIP-seq peaks was performed using HOMER. We found that the top 5 DNA binding motifs belonged to the transcription factors RelA, TBP, CEBPA, CTCFL, and YY1 (Fig. 6a). The observation that RelA (also called NF-κB p65) is essential for RANK/RANKL signaling and is a known binding partner of KDM4 subfamily members^4,48–50^ suggests that KDM4B could be a coactivator of p65. To investigate this possibility, we performed assays to detect the physical interaction between KDM4B and p65. Whole-cell extracts from 293T cells transiently expressing FLAG-KDM4B and HA-p65 were immunoprecipitated using an anti-FLAG antibody. Western blot analysis showed a strong interaction of KDM4B with p65 (Fig. 6b, top). As c-Fos and Nfatc1 are also key transcription factors involved in RANK-RANKL signaling supporting osteoclast differentiation,^4,5^ we next determined whether KDM4B is associated with these factors. Co-IP experiments revealed that KDM4B selectively associates with p65 but not with c-Fos or Nfatc1 (Fig. 6b, middle and bottom). To further determine the interacting domains of KDM4B and p65, we performed additional co-IP experiments using a set of KDM4B and p65 mutants. We found that the JmjC domain of KDM4B was necessary for interacting with the N-terminal domain of p65 (Fig. 6c, d). Additionally, our finding that the binding of a catalytically inactive KDM4B mutant to p65 suggests that the p65–KDM4B interaction is demethylase activity independent (Supplementary Fig. 6a). To support these data, we performed additional ChIP-seq experiments with p65 after RANKL treatment and analyzed the colocalization of KDM4B and p65 by comparing the ChIP-seq data sets. These results clearly showed that the p65 peaks were primarily colocalized with those of the KDM4B–CCAR1–MED1 complex in the Cluster III genes shown in Fig. 3c (Fig. 6e). We observed similar results upon intersecting the KDM4B profile with published ChIP-seq data sets (p65 enrichment after LPS treatment) (Supplementary Fig. 6b).

We next sought to examine whether the occupancy of KDM4B and p65 on chromatin is interdependent. ChIP assays demonstrated that p65, as well as KDM4B, CCAR1, and MED1, was highly enriched in the promoters of the *Fosl2* and *Tpm1* genes in response to RANKL treatment (Figs. 5b and 6f, control shRNA). Unexpectedly, the promoter localization of KDM4B did not decrease significantly after depletion of p65 (Fig. 6f and Supplementary Fig. 6c). We then investigated the effect of the KDM4B–CCAR1–MED1 axis on p65 localization. Interestingly, knockdown of KDM4B or CCAR1 reduced the p65 levels in the *Fosl2* and *Tpm1* promoters (Fig. 6g). We observed that individual depletion of KDM4B or CCAR1 did not affect RANKL-induced nuclear translocation of p65 (Supplementary Fig. 6d, e). Additionally, CCAR1 was not found to directly interact with p65 (Supplementary Fig. 6f). Based on these results, we hypothesize that KDM4B, in concert with CCAR1, demethylates H3K9me3 in the promoter regions of target genes and allows opening of the chromatin structure, thus facilitating access by p65.

### Pharmacological inhibition of the demethylase activity of KDM4B abolishes p65-mediated osteoclastogenesis and ameliorates ovariectomy-induced bone loss in ovariectomized (OVX) mice

**Fig. 7:**
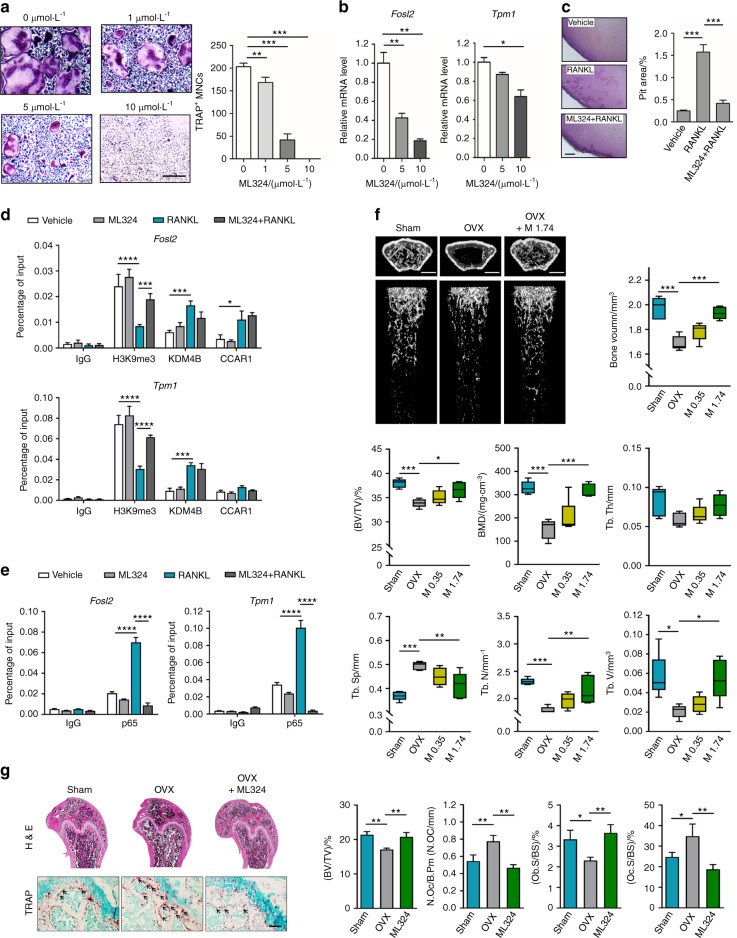
Administration of KDM4B inhibitors ameliorates bone loss in the ovariectomized (OVX) mouse model. **a** TRAP staining of BMMs treated with ML324. Scale bar, 75 μm. **b** mRNA expression levels of *Fosl2* and *Tpm1* in the cells evaluated in (**a**). **c** BMMs were treated with vehicle or 10 μmol·L^−1^ ML324 in the presence or absence of RANKL (100 ng·mL^−1^) for 10 days. The dentin slices were stained with Mayer’s hematoxylin, the resorption pit area was analyzed. Scale bar, 200 μm. **d**, **e** ChIP assay of H3K9me3, KDM4B, CCAR1, and p65 occupancy at the *Fosl2* promoter or *Tpm1* promoter in BMMs treated with ML324 (10 μmol·L^−1^) and/or RANKL for 30 min. **f** Micro-CT analysis of the femurs of 4-month-old sham-operated, saline-treated (Sham), saline-treated OVX (OVX), and ML324-treated OVX (OVX + M 1.74) mice (*n* = 5). Left panel, representative micro-CT image of a proximal femur (top, axial view; bottom, longitudinal view). Right panel, bone parameters. M 0.35, 0.35 mg·kg^−1^ body weight; M 1.74, 1.74 mg·kg^−1^ body weight. Scale bars, 1 mm. **g** Histological analysis of femur sections from the mice evaluated in (**f**) (*n* = 3). ML324, 1.74 mg·kg^−1^ body weight. The sections were stained for H&E and TRAP. The arrows indicate osteoclasts. Scale bars, 100 μm. The data are presented as the mean ± SD values of three independent experiments [one-way ANOVA in (**a**–**c**) and two-way ANOVA in (**d**, **e**)]. The data in (**f**, **g**) are presented as the mean ± SEM values (one-way ANOVA). *P < 0.05; **P < 0.01; ***P < 0.001. See also Supplementary Figs. 7 and 8

To further evaluate the demethylase activity of KDM4B on p65-induced osteoclast differentiation, two potent KDM4B inhibitors, ML324 and NSC636819, were employed. We first evaluated the effects of these inhibitors in suppressing RANKL-dependent osteoclastogenesis. Both ML324 and NSC636819 significantly inhibited osteoclast formation in a dose-dependent manner (Fig. 7a and Supplementary Fig. 7a), with concomitant reductions in the mRNA levels of KDM4B and NFATc1 target genes (Fig. 7b and Supplementary Fig. 7b–d). To examine the effect of Kdm4b demethylase activity on the bone resorption ability of osteoclasts in vitro, a pit formation assay was performed using OCPs. As shown in Fig. 7c, ML324 treatment significantly reduced RANKL-induced pit formation. Furthermore, reporter assays using wild-type KDM4B or its catalytically inactive mutants clearly showed that the transactivation activity of p65 is regulated by both the demethylase activity of KDM4B and CCAR1 (Supplementary Fig. 7e). We next investigated whether the demethylase activity of KDM4B can regulate p65 occupancy at KDM4B target genes. ChIP analysis of the *Fosl2* and *Tpm1* loci showed that RANKL treatment reduced the H3K9me3 levels but RANKL and ML324 cotreatment abrogated the decrease in H3K9me3 induced by RANKL alone (Fig. 7d, H3K9me3). Conversely, the sharp increase in p65 occupancy upon RANKL treatment was abrogated in the presence of ML324 without affecting p65 shuttling (Fig. 7e and Supplementary Fig. 7f). Moreover, the localization of KDM4B and CCAR1 at target genes was not affected by ML324 treatment (Fig. 7d, KDM4B and CCAR1). Consistent with the results of the depletion studies (Supplementary Fig. 5b), the global H3K9me3 levels were not changed upon combination treatment with RANKL and ML324 (Supplementary Fig. 7g). Collectively, these data further support our model proposing that the demethylase activity of KDM4B is critical in fine-tuning the recruitment and function of p65 during osteoclast differentiation.

Finally, we investigated the potential therapeutic efficacy of ML324 for osteoporosis in vivo. In the OVX mouse model, mice were administered two different doses of ML324 (Supplementary Fig. 8a). In evaluating the pharmacokinetic properties of ML324, we found that it exhibited an extremely large steady-state volume of distribution (146 L·kg^−1^) and a long half-life (10.8 h), demonstrating the good tissue distribution and long duration of the drug in the body (Supplementary Fig. 8b and Supplementary Table 1). No physiological differences (e.g., body weight) between the ML324-treated and vehicle-treated OVX groups were observed (Supplementary Fig. 8c). However, micro-CT analysis showed that BMD was significantly decreased in the OVX mice, while it increased gradually in ML324-injected OVX mice (Fig. 7f). We further examined the effect of ML324 on trabecular osteoclast numbers by bone histomorphometric analysis. ML324-injected OVX mice displayed reduced TRAP-positive staining compared to OVX mice (Fig. 7g). To correlate the ML324 antiosteoporotic activity of ML234 with KDM4B inhibition, bones from sham-, OVX-, and ML324-treated OVX mice were analyzed by immunohistochemistry. The levels of H3K9me3 were decreased in OVX mice compared to sham mice. However, H3K9me3 levels were restored after ML324 treatment (Supplementary Fig. 8d). These results indicate that ML324 displays antiosteoporotic activity by interfering with KDM4B-mediated osteoclastogenesis.

## Discussion

While there is increasing evidence for the role of histone demethylases in osteoblast differentiation, the role of histone demethylases in osteoclast formation is only now being addressed. In this study, we revealed an unprecedented role for KDM4B in regulating NF-κB-mediated osteoclast differentiation and bone homeostasis. Using conditional knockout mice, we found that KDM4B is essential for RANKL-induced osteoclastogenesis. Biochemical and ChIP-seq analyses demonstrated that the KDM4B–CCAR1–MED1 complex was physically and functionally required for NF-κB localization to the promoter regions of target genes. Moreover, studies of pharmacological inhibition of KDM4B provided compelling evidence that the KDM4B–CCAR1–MED1 complex functions as a key modulator of bone homeostasis by regulating osteoclastogenesis.

Our microarray and signaling pathway analyses demonstrated that KDM4B expression is transcriptionally regulated by RANKL-mediated JNK signaling. It has been reported that *Kdm4b* is transcriptionally regulated by several transcription factors, including ERα, AR, HIF-1α, and p53.^38,46,51,52^ Unexpectedly, our ChIP-seq data showed that p65 and KDM4B were enriched at the *Kdm4b* promoter region (Supplementary Fig. 9a). Moreover, knockdown of p65 reduced *Kdm4b* expression (Supplementary Fig. 9b). These results suggest that the RANKL-JNK-NF-κB-KDM4B signaling axis might be a key regulator of KDM4B expression. We observed that KDM4B was degraded by the ubiquitin-proteasome system but was rapidly stabilized after RANKL treatment. Recent studies have shown that Fbxo22 and Hsp90 mediate KDM4B stability by regulating its ubiquitylation.^53,54^ Thus, it is tempting to speculate that these factors regulate KDM4B protein levels during osteoclast differentiation. These findings support the idea that KDM4B, the expression of which is controlled at the mRNA and protein levels, can act as a key initiator of osteoclast differentiation.

We also observed that KDM4B-deficient OCPs failed to differentiate into osteoclasts and that conditional knockout mice with myeloid-specific *Kdm4b* deletion showed elevated bone mass associated with a decreased number of osteoclasts. Our unpublished data revealed that deletion of *Kdm4b* in osteoblasts accelerated osteoblast differentiation. Consistent with this finding, osteoblast-specific *Kdm4b*^cKO^ mice exhibited increased bone mass, similar to myeloid-specific *Kdm4b*^cKO^ mice (data not shown). These data are somewhat inconsistent with a recent report by Ye et al.^23^, which concluded that KDM4B promotes osteogenic differentiation of human MSCs. These differences can be explained by the distinct functions of KDM4B according to the stage of osteogenic differentiation, for example, (1) lineage commitment and progression (differentiation of MSCs into transitory osteoblasts; cell fate determination; Ye et al.^23^) and (2) differentiation and maturation (differentiation of transitory osteoblasts into osteoblasts and osteocytes; osteoblastogenesis; our study). In addition, Qi et al.^55^ recently reported that ML324 treatment promoted osteoblast differentiation. Although further investigation is needed to determine the specific mechanism by which KDM4B integrates multiple cellular signals to affect different biological processes, we hypothesize that KDM4B is a negative regulator of bone formation.

As previous studies showed that KDM4B cooperates with other proteins (e.g., MLL2 and SWI/SNF complexes) to function as a coactivator, it seemed likely that KDM4B-mediated osteoclastogenesis might be dependent on interactions with other factors.^30^ Using a series of column chromatographic purification and mass spectrometric analyses, we purified several interacting partners associated with KDM4B and characterized CCAR1 as a factor required for KDM4B-mediated osteoclastogenesis. CCAR1 physically interacted with KDM4B, and the pattern of CCAR1 expression was very similar to that of KDM4B. Knockdown of CCAR1 inhibited RANKL-induced osteoclastogenesis, and KDM4B–CCAR1 colocalized to the promoter regions of osteoclast-related genes. CCAR1 was also required for KDM4B-mediated H3K9 demethylase activity and functioned as a bridge between KDM4B and the Mediator complex. It is still unclear how CCAR1 regulates the demethylase activity of KDM4B. We observed that CCAR1 had no effect on KDM4B occupancy at the promoter regions of target genes (Fig. 5b). Given that histone-modifying enzymes often work in concert with other modifiers, transcription factors, and the transcriptional machinery, one possible explanation is that CCAR1 might be a core subunit supporting the demethylase activity of KDM4B in the nucleosome. It would be interesting to investigate the role of CCAR1 in the enzymatic function of KDM4B in greater detail. Interestingly, we found that the NURD complex was a KDM4B-interacting partner. As our unpublished results showed that knockdown of the NURD complex increased osteoclast formation, we postulated that the NURD complex may negatively regulate functions of KDM4B. Additional studies will be needed to detail the function of the NURD complex in osteoclastogenesis.

De novo motif analysis with the KDM4B ChIP-seq results suggested that p65 was a possible activator of KDM4B. We subsequently confirmed that KDM4B physically and functionally interacted with p65. Interestingly, knockdown and chemical perturbation of KDM4B showed that p65 localization is dependent on the demethylase activity of KDM4B. Recently, Wu et al.^26^ reported that KDM4B associated with c-Jun and that depletion of KDM4B significantly reduced c-Jun occupancy at specific promoters. Another group also demonstrated that KDM4B depletion resulted in decreased occupancy of SMAD3 at the SOX9 promoter.^34^ We suggest that KDM4B-mediated H3K9 demethylation shifts the structure of DNA from heterochromatin to euchromatin, which allows activators to bind their target promoters.

Based on our observations and previous reports, we constructed a working model explaining how KDM4B induces RANKL-dependent osteoclastogenesis (Supplementary Fig. 10). Initially, RANK-RANKL signaling increases the KDM4B protein level by transcriptional and posttranslational regulation. KDM4B then localizes to target genes. After binding to DNA, KDM4B recruits the CCAR1-Mediator complex through binding at the JmjC domain. Subsequent H3K9 demethylation then facilitates euchromatinization, which is followed by p65 enrichment at these promoters, thereby potentiating osteoclast-related gene transcription. Our results demonstrate that H3K9 demethylation is a promising therapeutic target for osteoporosis. Thus, disrupting the KDM4B–CCAR1 interaction or the intrinsic demethylase activity of KDM4B could be a novel strategy for treating various bone disorders.

## Methods

### Plasmids, antibodies, and reagents

FLAG-KDM4B WT and deletion mutant cDNA sequences in pCDEF were kindly provided by Dr. Okada.^30^ The CCAR1 cDNA sequence in pSG5 was kindly provided by Dr. Kim.^43^ To generate the HA-CCAR1 and HA-p65 deletion constructs, the corresponding cDNAs were amplified by PCR and inserted into pSG5 and pcDNA3.1, respectively. For mammalian expression of HA-c-Fos and HA-NFATc1, the corresponding cDNAs were amplified and ligated into pSG5 or pMX. Further details of plasmid construction are available upon request. Antibodies specific for NFATc1 (sc-7294), NF-κB p65 (sc-8008), and KDM4B (sc-374241) were purchased from Santa Cruz Biotechnology. Antibodies specific for KDM4B (A301-478A), CCAR1 (A300-435A), or MED1 (A300-793A) were obtained from Bethyl Laboratories. An anti-FLAG antibody was purchased from Sigma-Aldrich (SAB4200071), and an anti-HA-peroxidase antibody was purchased from Roche (12013819001). An anti-β-Actin antibody was purchased from Sino Biological (100166-MM10), and an anti-H3K9me3 antibody was purchased from Active Motif (39161). ML324 was purchased from Sigma-Aldrich (SML0741). NSC636819 was purchased form Tocris (5287).

### Cell culture

HEK293T cells (ATCC) originating from a human fetal kidney and HeLa S3 cells (ATCC) originating from the female cervix were cultured in DMEM (Corning) supplemented with 10% FBS (Merck) and 1% penicillin-streptomycin (Corning).

To isolate primary OCP cells, bone marrow cells were collected from femurs and tibias of 6–8-week-old C57BL/6 mice and cultured in α-minimum essential medium supplemented with 10% FBS and M-CSF (5 ng·mL^−1^) for 16 h. Nonadherent cells were harvested and further cultured with M-CSF (30 ng·mL^−1^) for 3 days. After removal of floating cells, adherent cells were used as BMM cells.

### Mice

Mice were maintained in accordance with the Institutional Animal Care and Use Committees of Chungbuk National University. For all experiments, the mice were on a C57BL/6J background. All mice were housed in a mouse facility on a 12‐h light/dark cycle in a 22 °C temperature‐controlled room. Floxed Kdm4b (Kdm4b^*flox/flox*^) mice were kindly provided by Dr. Okada’s laboratory.^30^ Lyz2‐Cre transgenic mice were obtained from The Jackson Laboratory (Bar Harbor, ME, USA). To generate myeloid-specific *Kdm4b* knockout mice, Kdm4b^*flox/flox*^ mice were crossed with mice expressing Cre recombinase under the control of the Lyz2 promoter. Age-matched male mice were used for experiments.

### Microarray analysis

To assess differential expression of histone demethylases during RANKL-induced osteoclastogenesis, gene expression microarray data (GSE57468) were analyzed using ArrayPipe software.

### Osteoclastogenesis and TRAP staining

BMMs were cultured in the presence of M-CSF (30 ng·mL^−1^) and RANKL (100 ng·mL^−1^). After 3–6 days, cells were fixed and stained for TRAP using an acid phosphatase leukocyte kit (Sigma, 386A). TRAP-positive multinucleated cells containing three or more nuclei were counted as osteoclasts under a light microscope.

### Real-time quantitative RT-PCR

Total RNA was prepared using Tri-RNA reagent (Favorgen, FATRR001), and cDNA was reverse transcribed using M-MLV reverse transcriptase (Promega, M1708) according to the manufacturer’s instructions. Real-time PCR was performed using IQ SYBR Green Supermix in an IQ5 real-time thermal cycler (Bio-Rad). Relative mRNA levels were normalized to β-actin mRNA levels. The primers used for PCR are described in Supplementary Table 2.

### Preparation of whole-cell lysates and subcellular fractionation

BMMs were incubated with MG132 (10 μmol·L^−1^) (Sigma, M8699) and/or ML324 (Sigma, SML0741) for 1 h before RANKL treatment. For preparation of whole-cell lysates, cells were lysed in SDS lysis buffer [20 mmol·L^−1^ Tris (pH 7.5), 50 mmol·L^−1^ NaCl, 2.5% SDS, 2.5% sodium deoxycholate, 0.5 mmol·L^−1^ PMSF, and protease inhibitors] and briefly sonicated. To obtain the cytosolic and nuclear fractions, cells were resuspended in buffer A [10 mmol·L^−1^ HEPES (pH 7.9), 10 mmol·L^−1^ KCl, 1.5 mmol·L^−1^ MgCl_2_, 0.34 mol·L^−1^ sucrose, 10% glycerol, 1 mmol·L^−1^ DTT, 0.1% Triton X-100, and protease inhibitors] and incubated on ice for 8 min. After centrifugation at 300 × *g* for 5 min at 4 °C, the supernatant (cytosolic fraction) was collected in a new tube. The nuclear pellet was washed once with buffer A, lysed with SDS lysis buffer and briefly sonicated (nuclear fraction).

### Lentivirus-mediated RNA interference

DNA oligonucleotides encoding shRNAs specific for *Kdm4b*, *Ccar1*, *Ccar2*, *Smarca4*, *Med1*, and *p65* were annealed and ligated into the lentiviral expression vector pLKO.1 (Addgene). Lentiviral particles were generated as previously described.^56^ For the knockdown study, BMMs were infected with these viruses and selected with puromycin (2 μg·mL^−1^) for 2 days. BMMs were then differentiated with M-CSF (30 ng·mL^−1^) and RANKL (100 ng·mL^−1^). The DNA oligonucleotides encoding the shRNAs are described in Supplementary Table 3.

### Bone resorption assay

BMMs were seeded on dentin slices (IDS, AE-8050), treated with or without 10 μmol·L^−1^ ML324 containing M-CSF (30 ng·mL^−1^) and RANKL (100 ng·mL^−1^), and further cultured for 10 days. The dentin slices were then ultrasonicated, and resorption pits on the slices were stained with Mayer’s hematoxylin (Sigma, MHS1). The areas of pits on the slices were analyzed using ImageJ software.

### Ovariectomy and micro-CT analysis

For ovariectomy experiments, 2-month-old female C57BL/6 mice (20 g) were divided randomly into three groups (*n* = 5): sham-operated mice, bilateral OVX mice treated with vehicle, and OVX mice treated with ML324. After the operation, mice were injected intraperitoneally with vehicle or ML324 (0.35 or 1.74 mg·kg^−1^ body weight, respectively) once a week for 8 weeks.

For micro-CT analysis, the distal femur was scanned using a Quantum GX micro-CT imaging system (PerkinElmer, Hopkinton, MA, USA), and trabecular bone parameters were analyzed by Analyze 12.0 software (AnalyzeDirect, Overland Park, KS, USA).

### Bone histomorphometry

For hematoxylin and eosin (H&E) and TRAP staining, the femurs of mice were excised, fixed with 4% paraformaldehyde solution, and decalcified in 0.5 mol·L^−1^ ethylenediaminetetraacetic acid (EDTA, pH 7.4). The femurs were then dehydrated with ethanol, cleared with xylene, and embedded in paraffin. The femur sections (5 μm thick) were stained with H&E and TRAP. Histological examination was carried out as previously described.^57^

### Identification of KDM4B-interacting proteins

Nuclear extracts were prepared from HeLa S3 cells expressing FLAG-KDM4B and initially fractionated by Q-Sepharose chromatography (GE Healthcare, 17-0510-01). The fractions containing ectopic KDM4B were combined and subjected to sequential column chromatography on heparin Sepharose (GE Healthcare, 17-0998-01), DEAE Sepharose (GE Healthcare, 17-0709-01), and M2 agarose (Sigma, A220) columns. The proteins copurified with KDM4B were analyzed by tandem mass spectrometry.

### Immunoprecipitation

For immunoprecipitation assays, 293T cells were transfected with HA-NFATc1, HA-c-FOS, or HA-p65 together with FLAG-KDM4B WT or FLAG-KDM4B mutants. Cell lysates were prepared by lysis with NP-40 lysis buffer [10 mmol·L^−1^ Tris (pH 7.9), 150 mmol·L^−1^ NaCl, 1 mmol·L^−1^ EDTA, 5% glycerol, and protease inhibitors] and subjected to immunoprecipitation with an anti-FLAG antibody (Sigma, SAB4200071); the bound proteins were then analyzed by immunoblotting with an anti-HA antibody. For co-IP of endogenous proteins, BMMs stimulated with RANKL (100 ng·mL^−1^, 30 min) were immunoprecipitated using an anti-KDM4B antibody (Santa Cruz, sc-374241) and immunoblotted with an anti-CCAR1 antibody (Bethyl Laboratories, A300-435A).

### ChIP-sequencing

Cells were crosslinked with 1% formaldehyde for 10 min and washed with ice-cold PBS. The crosslinked cells were lysed with hypotonic buffer [10 mmol·L^−1^ HEPES-KOH (pH 7.8), 10 mmol·L^−1^ KCl, 1.5 mmol·L^−1^ MgCl_2_, and protease inhibitors] on ice for 10 min and centrifuged for 1 min at 14 000 r·min^−1^. The nuclear pellet was resuspended in nuclear lysis buffer [1% SDS, 50 mmol·L^−1^ Tris-HCl (pH 8.0), 0.5 mmol·L^−1^ EDTA, and protease inhibitors] for 1 h and sonicated using a Bioruptor (Diagenode) for 20 cycles. After preclearing, ChIP assays were performed using antibodies specific for KDM4B (Bethyl Laboratories, A301-478A), CCAR1 (Bethyl Laboratories, A300-435A), MED1 (Bethyl Laboratories, A300-793A), p65 (Santa Cruz, sc-8008), and H3K9me3 (Active Motif, 39161). Immunoprecipitated DNA was subjected to DNA sequencing (e-biogen, Korea). Sequencing reads were aligned to the reference mouse genome (mm10 assembly) using HISAT2 with default parameters, and identical reads were removed from further analysis. A minimum of 10 million uniquely mapped reads were obtained for each condition. We used the makeTagDirectory command followed by the findPeaks command in HOMER version 4.9.1 to identify enriched ChIP-seq peaks with respect to the background. A false discovery rate (FDR) threshold of 0.001 was used for all data sets. The total number of mapped reads in each sample was normalized to 10 million mapped reads. ChIP-seq data were visualized by preparing custom tracks for the UCSC Genome browser. To find GO terms, we used the Metascape tool.

### Motif enrichment analysis

We performed de novo analysis on ±100 bp sequences centered on the ChIP-seq peak regions using the “findMotifsGenome.pl” command in the HOMER package. The peak sequences were compared to random genomic fragments of the same size and a normalized G + C content to identify motifs enriched in the targeted sequences.

### Statistical analysis

Data are presented as the mean ± SEM or the mean ± SD values. The exact numbers of replicates (*n*) are indicated in the figure legends. The significance of differences was evaluated using the two-tailed *t*-test or Kolmogorov–Smirnov test for comparisons between two groups and one-way ANOVA or two-way ANOVA followed by Tukey’s multiple comparison test for comparisons among three or more groups. A *P* value < 0.05 was considered significant. In the figures, the asterisks denote statistical significance (*P < 0.05; **P < 0.01; ***P < 0.001; ****P < 0.000 1). Statistical analysis was performed in GraphPad PRISM 8.

## Supplementary information

Supplementary Information
