# Supplementary material for: The KDM4B–CCAR1–MED1 axis is a critical regulator of osteoclast differentiation and bone homeostasis
Source: Bone Res. 2021 May 25;9:27. doi: 10.1038/s41413-021-00145-1 (PMC8144413; doi:10.1038/s41413-021-00145-1)
Supplement: Supplementary file 1 — Supplementary Information [file 41413_2021_145_MOESM1_ESM.docx]

**Supplementary Information**

**The KDM4B-CCAR1-MED1 axis is a critical regulator of osteoclast differentiation and bone homeostasis**

Sun-Ju Yi, You-Jee Jang, Hye-Jung Kim, Kyubin Lee, Hyerim Lee, Yeojin Kim, Junil Kim, Seon Young Hwang, Jin Sook Song, Hitoshi Okada, Jae-Il Park, Kyuho Kang, and Kyunghwan Kim

**
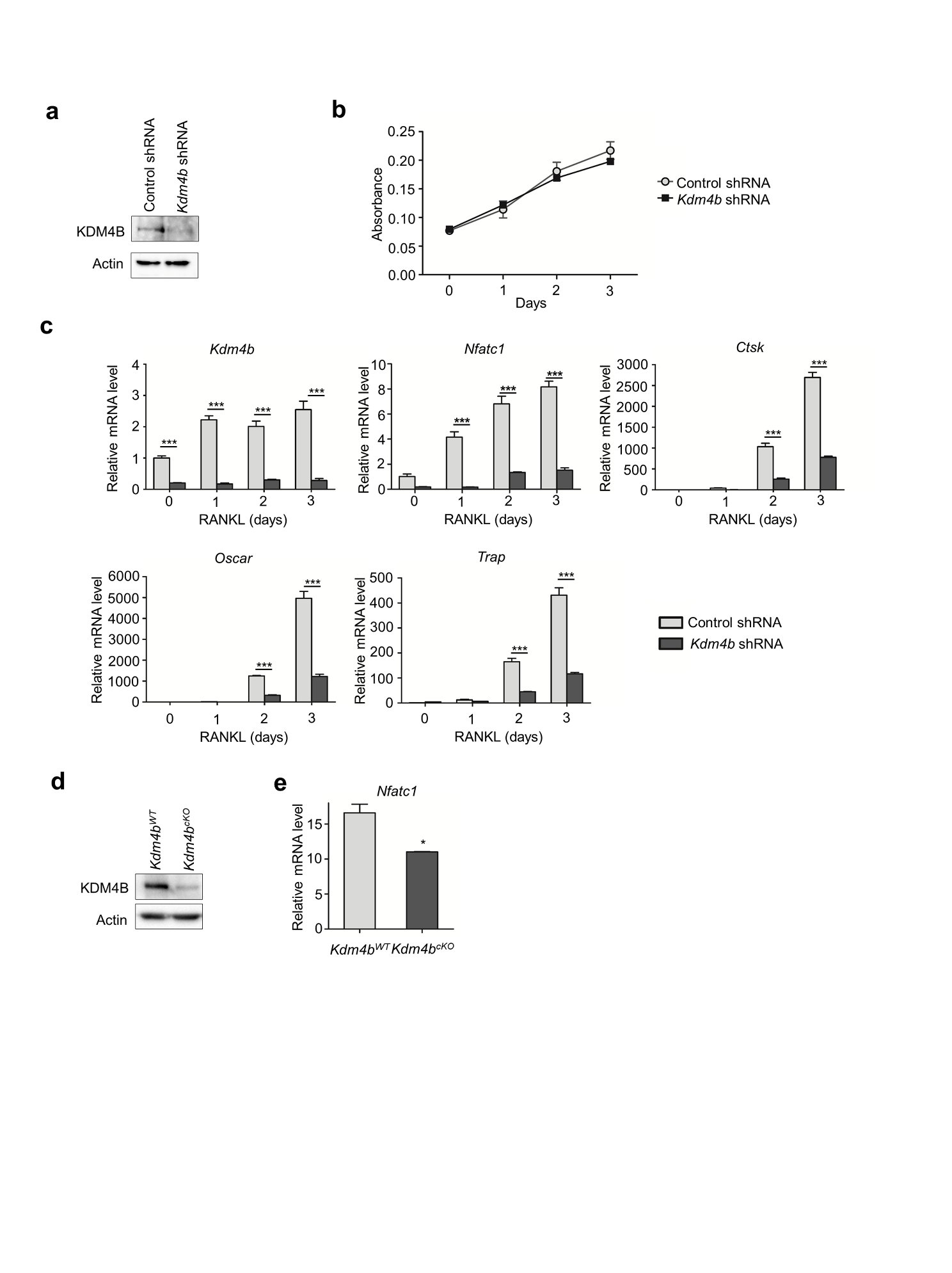
**

**Supplementary Fig.** **1 KDM4B is required for osteoclast differentiation.**

**a** Immunoblot of BMM lysates after knockdown of *Kdm4b*.

**b** Cell proliferation assay of Mock- or KDM4B-depleted BMMs.

**c** mRNA expression of *Nfatc1* and its target genes from Mock- or KDM4B-depleted BMMs as in **a**.

**d** Immunoblot of BMM lysates from *Kdm4b* osteoclast-specific conditional knockout mice.

**e** mRNA expression of *Nfatc1* from BMMs as in Fig. 1h.

Data represent mean result ± SD of three independent experiments (two-way ANOVA in **c**, two-tailed *t* test in **e**). *, *p* < 0.05; **, *p* < 0.01; ***, *p* < 0.001.

**
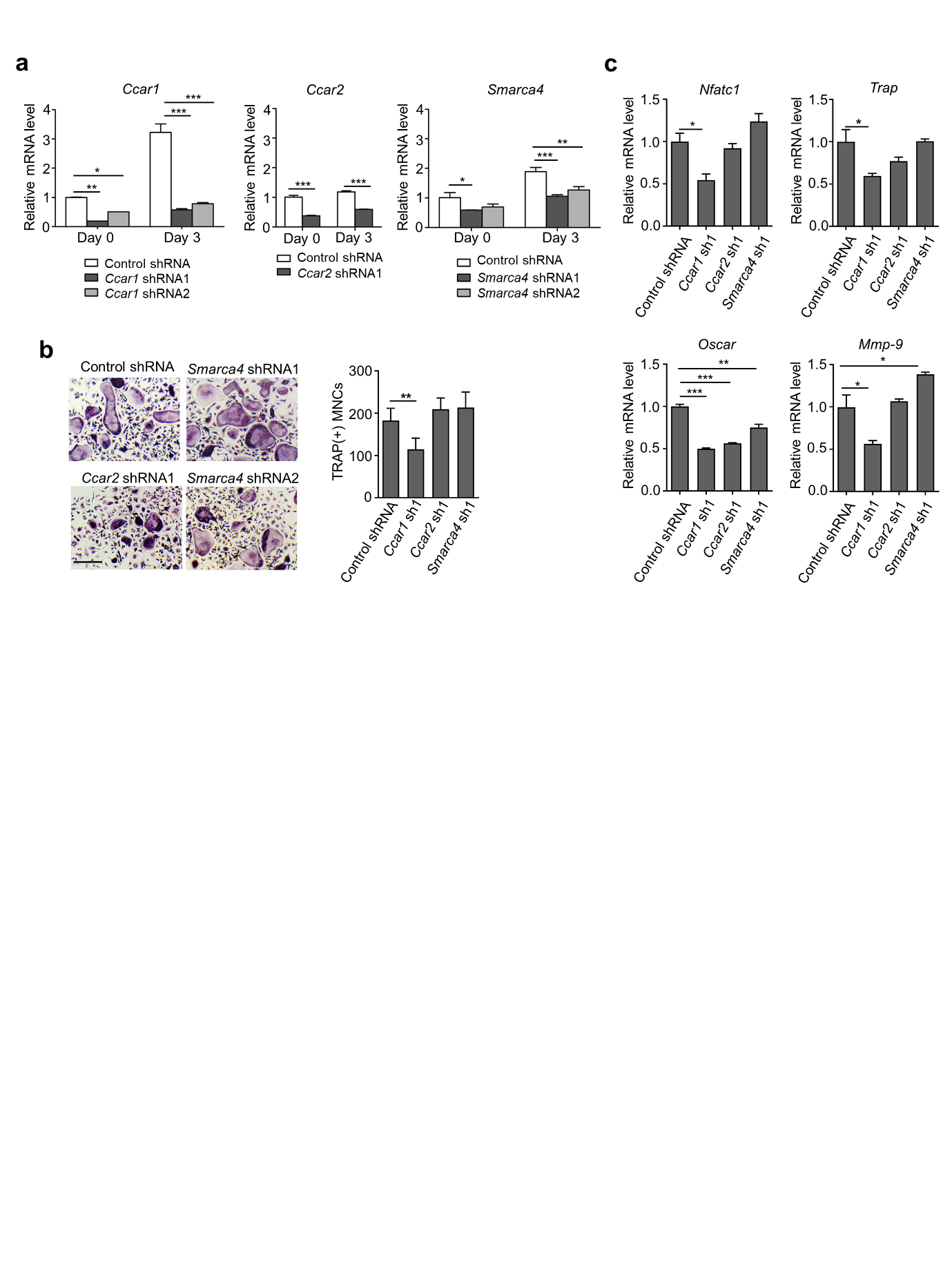
**

**Supplementary Fig. 2 CCAR1, one of KDM4B-interacting proteins, is necessary for osteoclastogenesis.**

**a** Validation of knockdown of *Ccar1*, *Ccar2* and *Smarca4* by qRT-PCR.

**b** TRAP staining of BMMs expressing control shRNA, *Ccar2* shRNA or *Smarca4* shRNA. Scale bar, 75 μm.

**c** mRNA expression of *Nfatc1* and its target genes from BMMs as in **b**.

Data represent mean result ± SD of three independent experiments (two-way ANOVA in **a**-**c**). *, *p* < 0.05; **, *p* < 0.01; ***, *p* < 0.001.


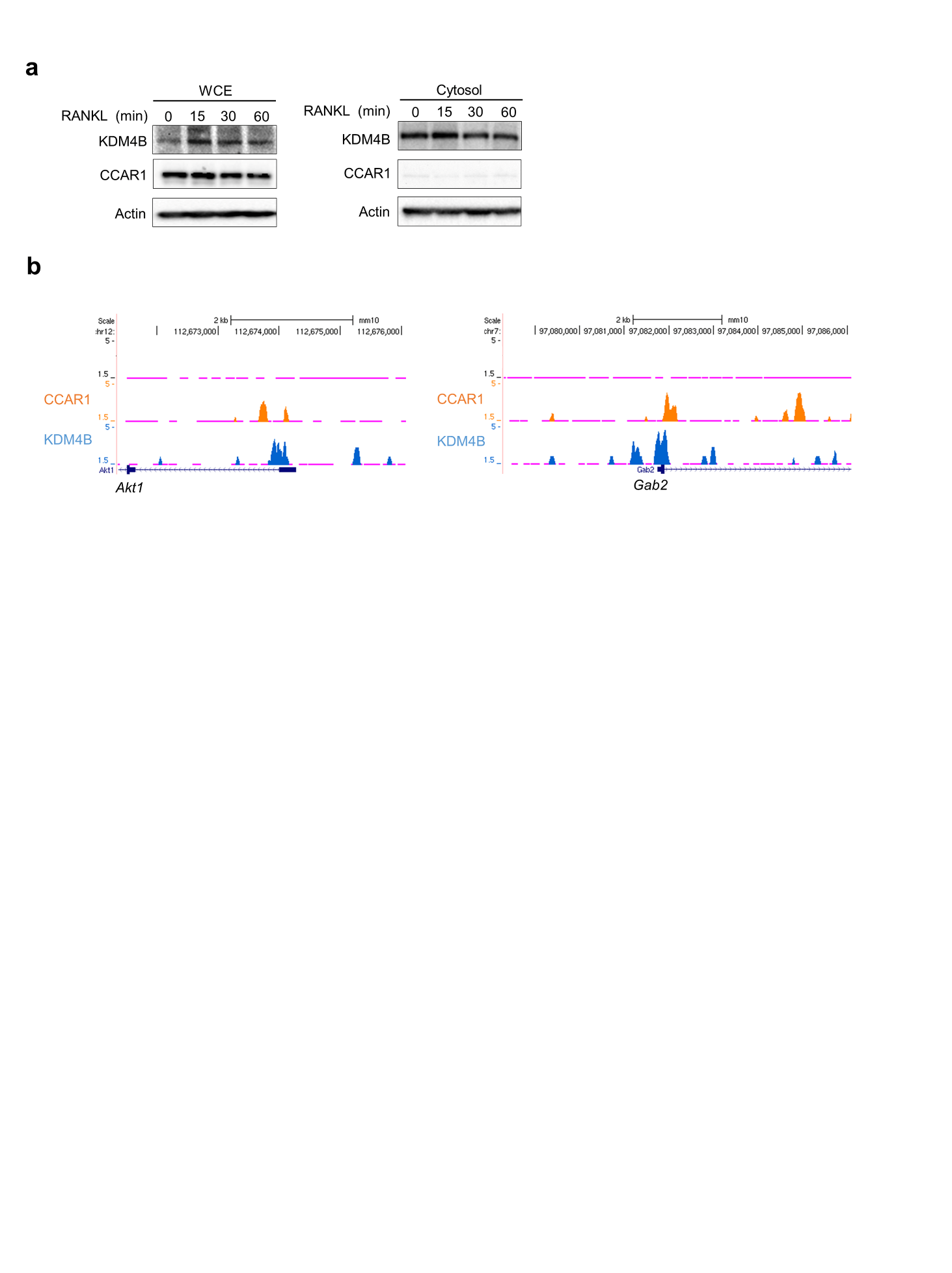


**Supplementary Fig. 3 Co-occupancy of KDM4B and CCAR1 to a set of osteoclast-related genes upon RANKL treatment.**

**a** Subcellular distribution of KDM4B and CCAR1 upon RANKL treatment. Immunoblots of whole cell lysates (WCE) and cytosolic fraction of BMMs upon RANKL treatment.

**b** Representative UCSC Genome Browser tracks showing KDM4B and CCAR1 co-occupancy at *Akt1* and *Gab2*.

**
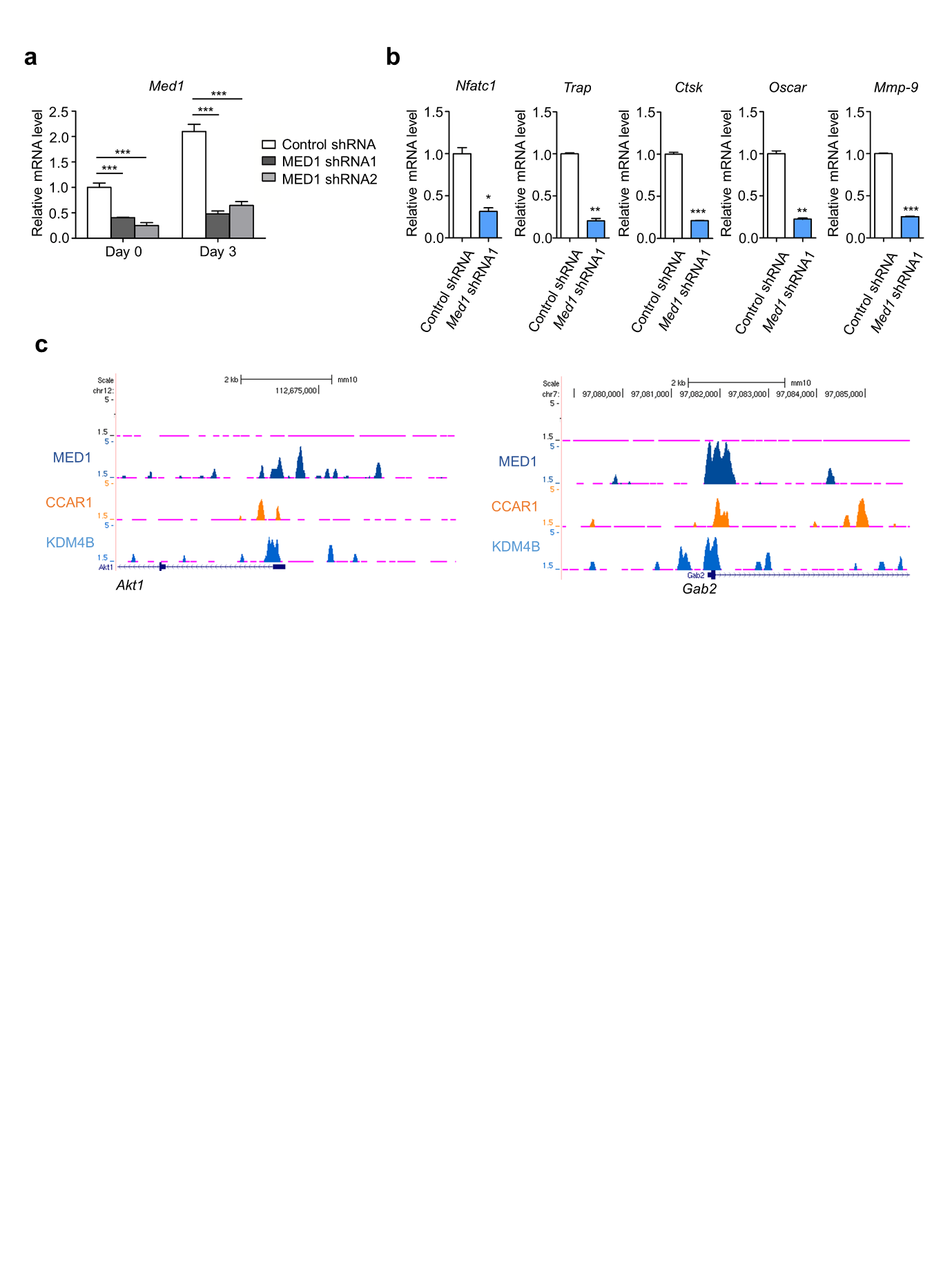
**

**Supplementary Fig. 4 KDM4B-CCAR1-MED1 axis modulates RANKL-induced osteoclastogenesis.**

**a** Validation of *Med1* knockdown from BMMs by qRT-PCR.

**b** mRNA expression of *Nfatc1* and its target genes from BMMs as in **a**.

**c** Representative UCSC Genome Browser tracks showing KDM4B, CCAR1 and MED1 co-occupancy at *Akt1* and *Gab2*.

Data represent mean result ± SD of three independent experiments (two-way ANOVA in **a**, two-tailed *t* test in **b**). *, *p* < 0.05; **, *p* < 0.01; ***, *p* < 0.001.


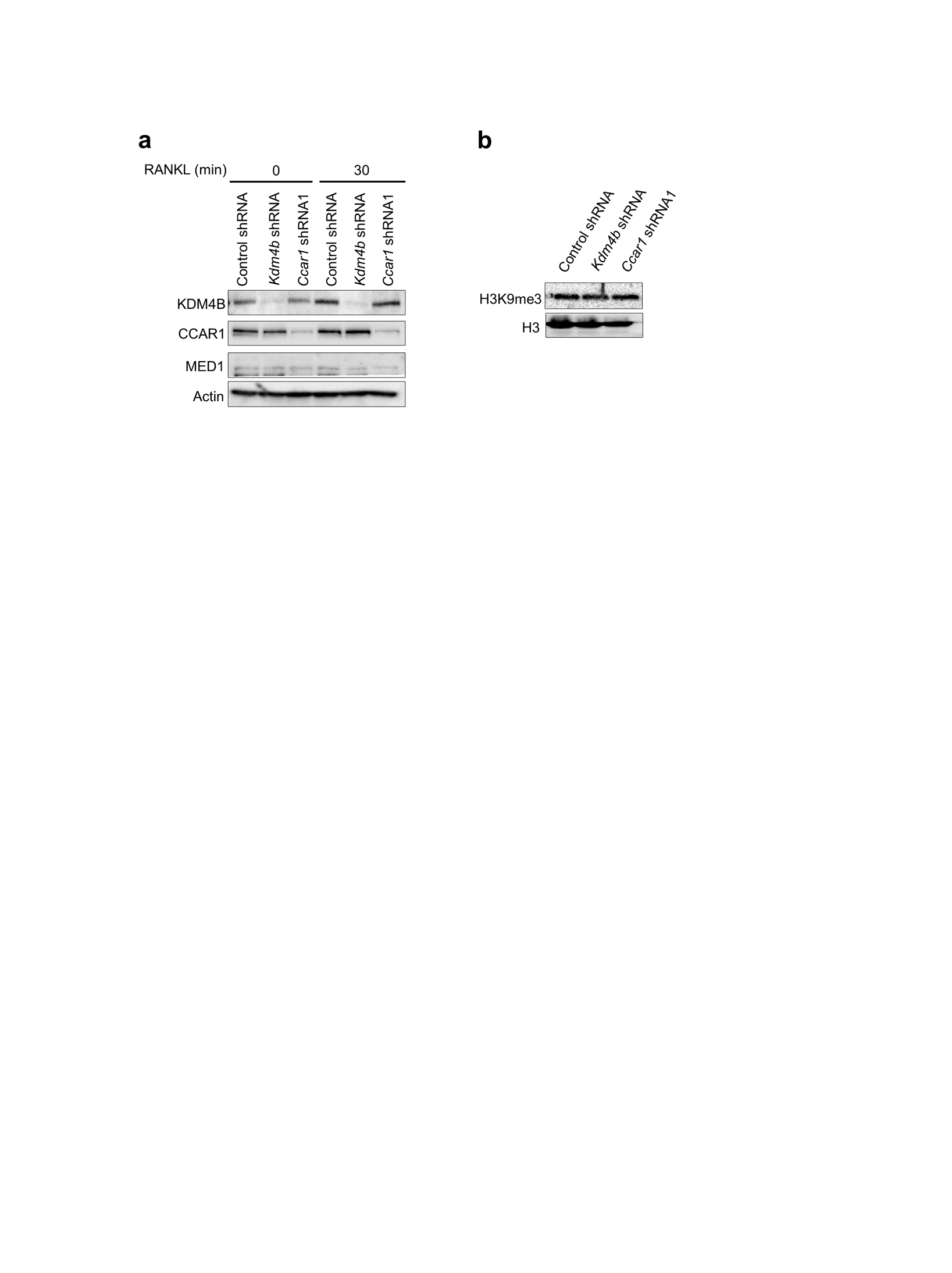


**Supplementary Fig. 5 Depletion of KDM4B or CCAR1 had little effect on global level of H3K9me3.**

**a** Effect of *Kdm4b* or *Ccar1* knockdown on protein expression of KDM4B, CCAR1, and MED1. Immunoblots of whole cell lysates of BMMs upon RANKL treatment ((100 ng/ml, 30 min).

**b** Global level of H3K9me3 in KDM4B- or CCAR1-depleted cells.

**
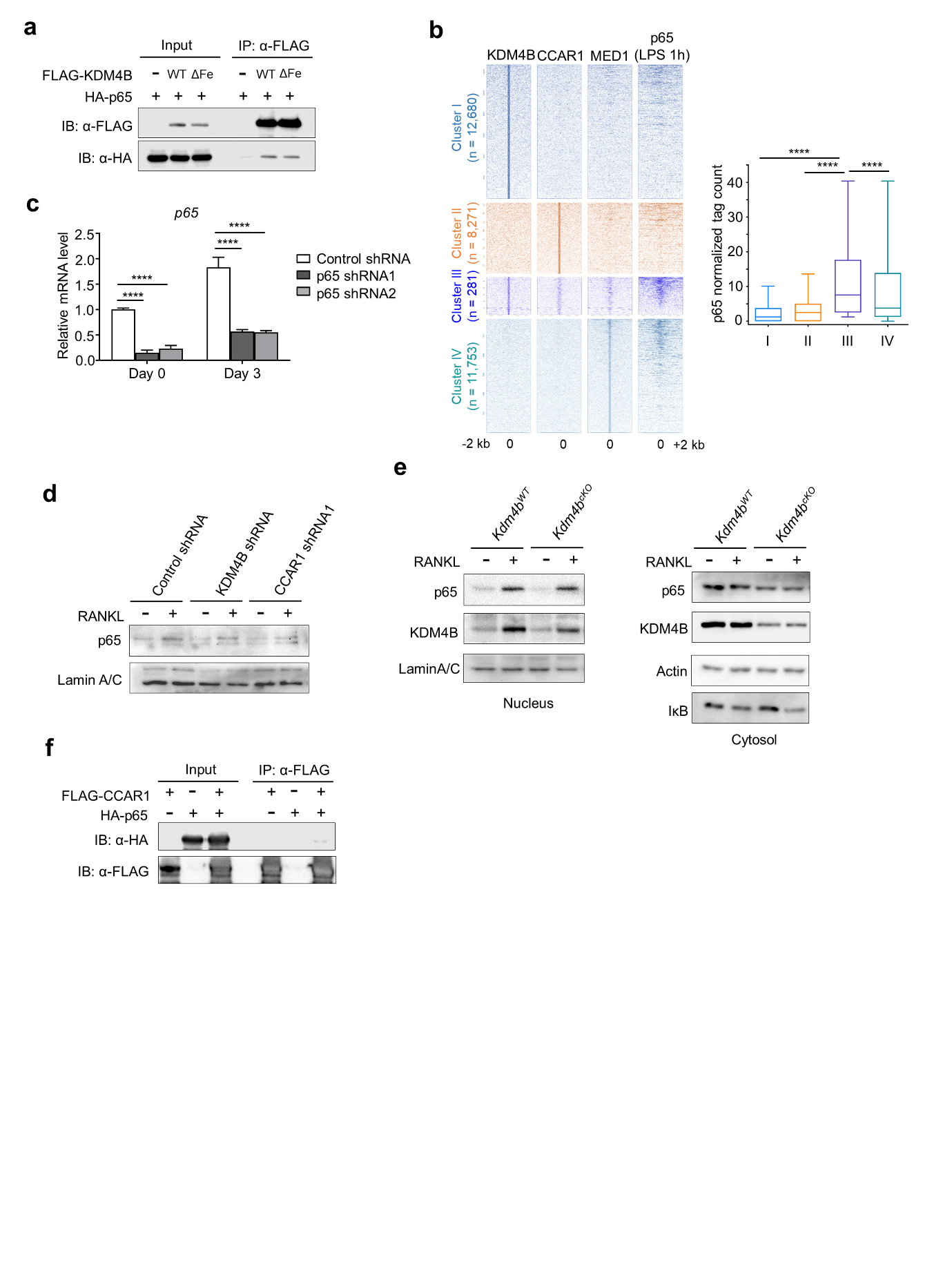
**

**Supplementary Fig. 6 p65 is an activator of KDM4B-CCAR1-MED1 axis.**

**a** Interaction with a KDM4B ΔFe (catalytically inactive form) and p65.

**b** Heatmap of normalized tag densities between KDM4B-CCAR1-MED1 (from this study) and p65 (LPS 1h) (from public data) peaks (left panels). Quantification of p65 tag counts shown in box plots (right panel). Boxes encompass the 25th and 75th percentile changes. Whiskers extend to the 10th and 90th percentiles. The central horizontal bar indicates the median. ****, *p* < 0.0001 (Kolmogorov-Smirnov test).

**c** Validation of knockdown of *p65* by qRT-PCR.

**d** Effect of *Kdm4b* or *Ccar1* knockdown on p65 nuclear localization upon RANKL treatment (100 ng/ml, 30 min).

**e** Effect of *Kdm4b* knockout on p65 distribution upon RANKL treatment (100 ng/ml, 30 min).

**f** Coimmunoprecipitation study of FLAG-CCAR1 and HA-p65.

**
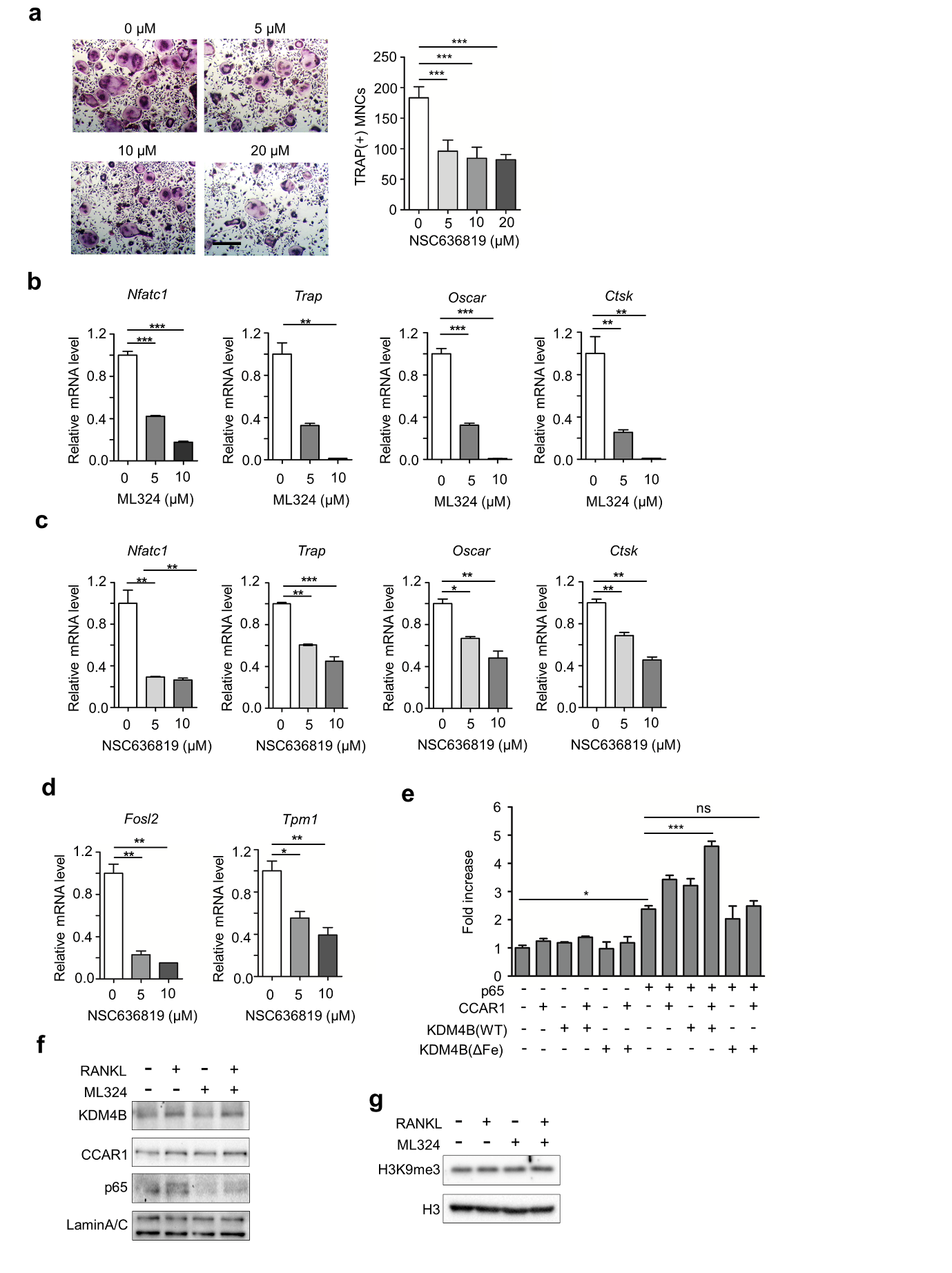
**

**Supplementary Fig. 7 KDM4B inhibitors, ML324 and NSC636819, suppress osteoclastogenesis.**

**a** TRAP staining of BMMs treated with RANKL (100 ng/ml) and NSC636819 for 3 days. Scale bar, 75 μm.

**b** mRNA expression of *Nfatc1* and its target genes from BMMs cells as in Fig. 7a.

**c** mRNA expression of *Nfatc1* and its target genes from BMMs cells as in **a**.

**d** mRNA expression levels of *Fosl2* and *Tpm1* in cells as in **a**.

**e** 293T cells were transiently transfected with the reporter plasmid *pNF-κB-Luc* along with *p65*, *Kdm4b* *WT*, *Kdm4b ΔFe*, or *Ccar1*. Luciferase activity was measured after 36 h post-transfection. Each bar represents the mean ± SD of three independent experiments.

**f** Effect of ML324 on p65 distribution upon RANKL treatment.

**g** Global level of H3K9me3 in cells as in **f**.

Data represent mean result ± SD of three independent experiments (one-way ANOVA in **a**-**e**). *, *p* < 0.05; **, *p* < 0.01; ***, *p* < 0.001.

**
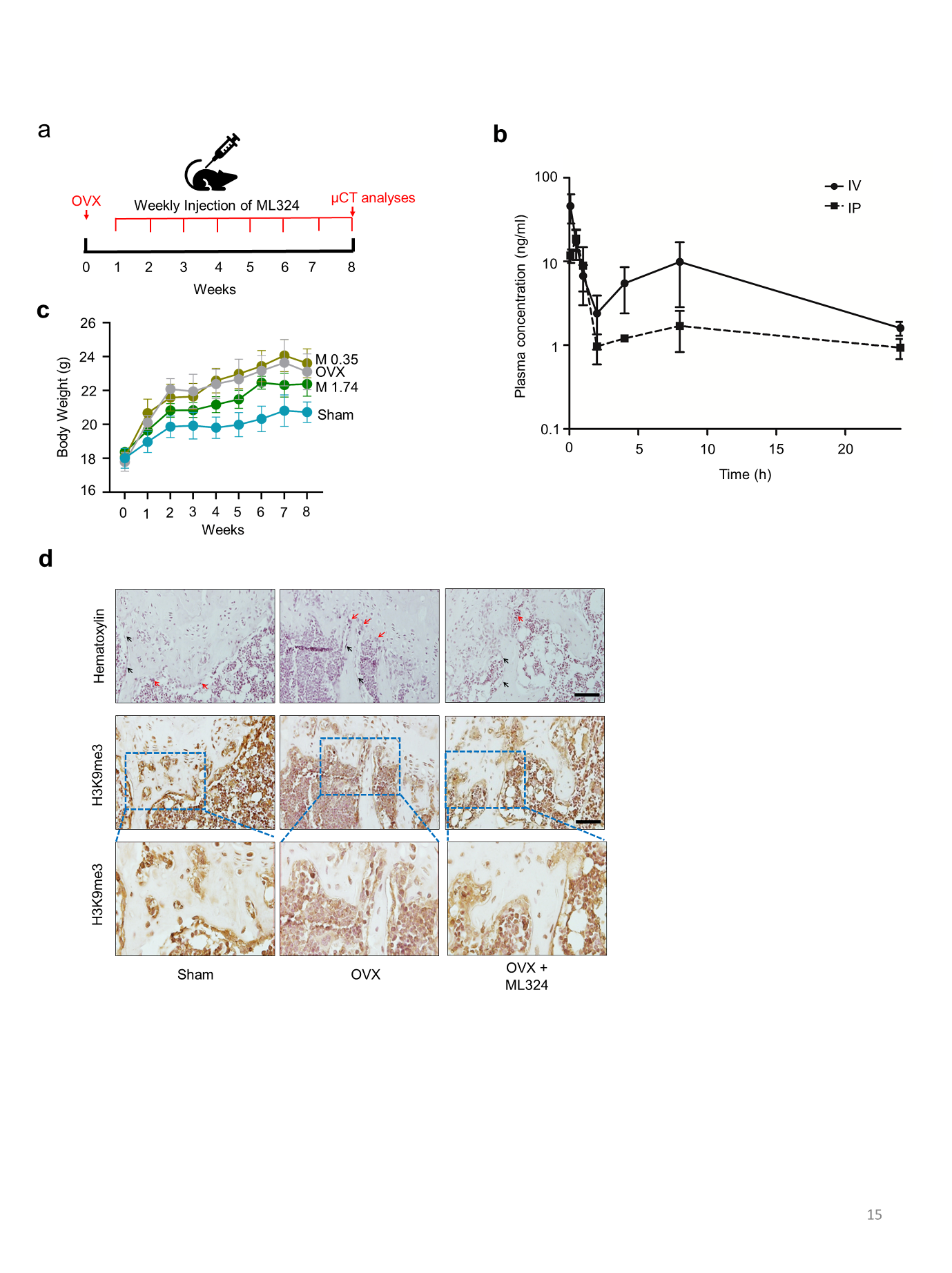
**

**Supplementary Fig. 8 Experimental procedure of ML324 injection in ovariectomized mice.**

**a** Schematic representation of the experimental design of the ovariectomized mouse study.

**b** Plasma concentration of ML324 after intravenous and intraperitoneal administration at 1.74 mg/kg in mice.

**c** Body weights of mice as in **a**. Data represent mean result ± SEM (n=5).

**d** Immunohistochemical staining of H3K9me3 in femur sections from Sham, OVX, and ML324-treated OVX. Boxed areas represent magnified images (bottom row). Red arrow: osteoclast, black arrow: osteoblast. Scale bar, 100 μm.

**
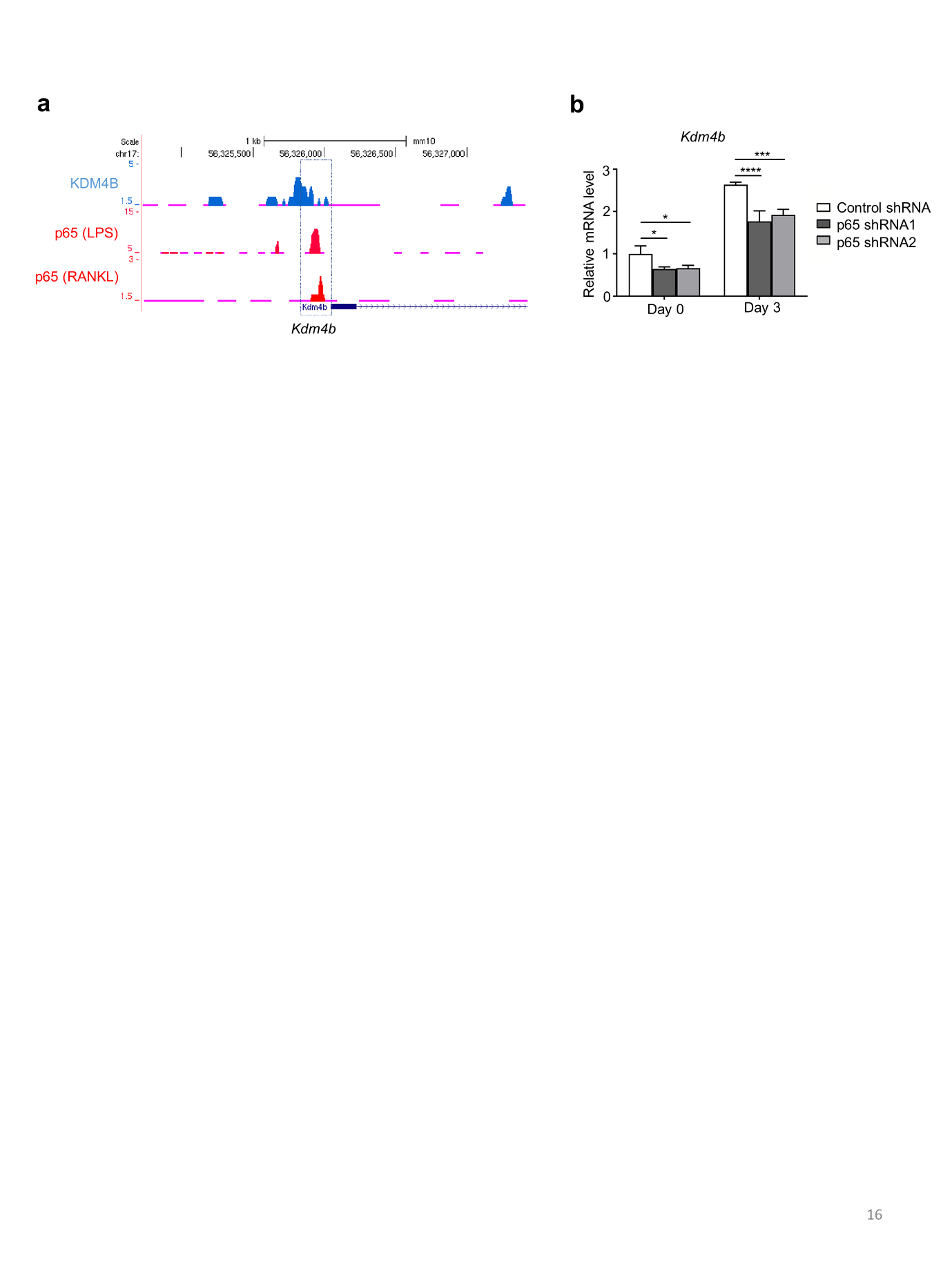
**

**Supplementary Fig. 9 p65 may cooperate with KDM4B to regulate *Kdm4b* mRNA expression**

**a** Representative UCSC Genome Browser tracks showing KDM4B and p65 co-occupancy at *Kdm4b*.

**b** Effect of *p65* knockdown on *Kdm4b* expression during osteoclast differentiation.

Data represent mean result ± SD of three independent experiments (one-way ANOVA in **b**). *, *p* < 0.05; **, *p* < 0.01; ***, *p* < 0.001.


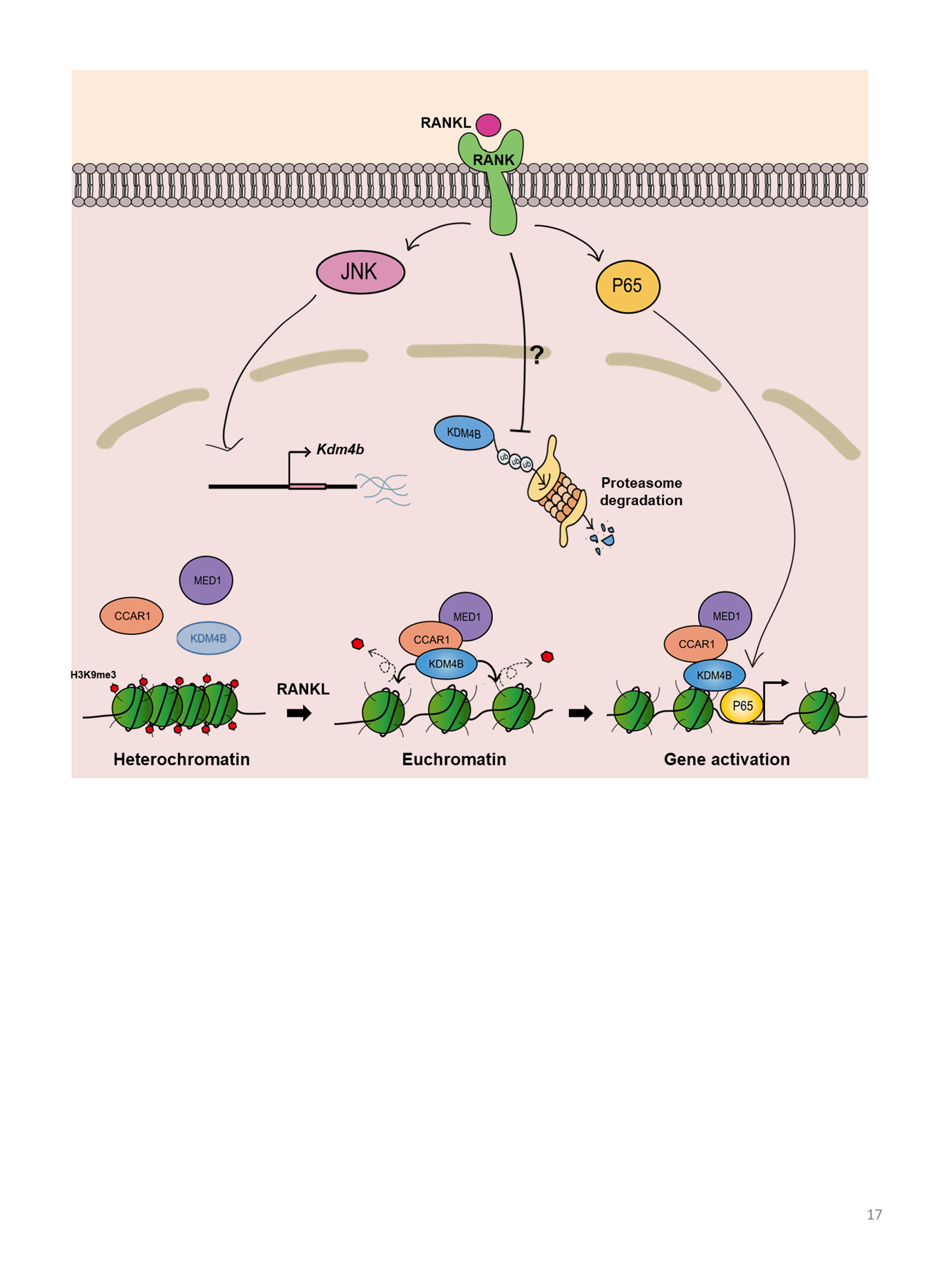


**Supplementary Fig. 10 Model for the cooperative role of KDM4B-CCAR1-MED1 axis in regulating p65-mediated osteoclastogenesis.** RANK-RANKL signaling upregulates *Kdm4b* mRNA level via JNK and inhibits KDM4B protein degradation, resulting in the increase of KDM4B protein level. Once KDM4B localizes to target genes, KDM4B recruits CCAR1-MED1 complex and subsequently demethylates H3K9me3, thereby shifting to euchromatin status. Then, p65 is recruitment to the promoters via KDM4B, which activates osteoclast-related gene expression.

**Supplementary Table 1** Pharmacokinetic parameters of ML324 after intravenous (IV) and intraperitoneal (IP) administration at 1.74 mg/kg in mice.

| **Mouse PK** | **IV** | **IP** |
| --- | --- | --- |
| Tmax (h) | - | 0.5 ± 0.00 |
| Cmax (µg/ml) | - | 0.02 ± 0.01 |
| AUC_t_ (µg/ml*h) | 0.16 ± 0.09 | 0.05 ± 0.02 |
| AUC_inf_ (µg/ml*h) | 0.19 ± 0.09 | 0.06 ± 0.02 |
| T_1/2_ (h) | 10.8 ± 3.7 | 9.83 ± 1.28 |
| CL (L/h/kg) | 11.1 ± 5.4 | - |
| V_ss_ (L/kg) | 146 ± 77.7 | - |
| F (%) | - | 29.7 |

The data shown are the means±S.D (n=3).

**Supplementary Table 2 Primers**

| **TARGET** | **Forward sequences (5’ → 3’)** | **Reverse sequences (5’ → 3’)** |
| --- | --- | --- |
| **RT-PCR** | | |
| *β-Actin* | GCAAGTGCTTCTAGGCGGAC | AAGAAAGGGTGTAAAACGCAGC |
| *Ctsk* | ACGGAGGCATTGACTCTGAAGATG | GGAAGCACCAACGAGAGGAGAAAT |
| *Nfatc1* | CTCGAAAGACAGCACTGGAGCAT | CGGCTGCCTTCCGTCTCATAG |
| *Oscar* | CTGCTGGTAACGGATCAGCTCCCCAGA | CCAAGGAGCCAGAACCTTCGAAACT |
| *Trap* | CTGGAGTGCACGATGCCAGCGACA | TCCGTGCTCGGCGATGGACCAGA |
| *Mmp-9* | CGTCGTGATCCCCACTTACT | AACACACAGGGTTTGCCTTC |
| *Kdm4a* | TTCTGTGAATCCTGCGTCTG | TAGCTGTGCACGGTGAGAAC |
| *Kdm4b* | CATGTGGAAGACCACGTTTG | AAGGGGATGCCGTACTTCTT |
| *Kdm4c* | GATGACTGGCCTTACGTGGT | CTTCACACAGTTTCGGCTCA |
| *Kdm4d* | TCACCGATTTATGGTGCTGA | GGTCTTCCACATGCCAAAGT |
| *Ccar1* | CGTTTGGTGTTCAGGCACAG | GTGGATCTAATCTCCGCGCA |
| *Ccar2* | AGCGAGTCTTCACAGGCATT | TCTGCACCTTGACAGCATTC |
| *Smarca4* | AAGGCCACCGCATGAAAAAC | CTTGTTCTGCAGTGGTGTGC |
| *P65* | GGAGTTCCAGTACTTGCC | GTCCTTTTGCGCTTCTCT |
| *Med1* | TCCTGTGACGATGAGGGCTA | CATTGGGTTGTTGCCTGTGG |
| *Fosl2* | ATCCACGCTCACATCCCTAC | GTTTCTCTCCCTCCGGATTC |
| *Tpm1* | GTATGAAGAGGTGGCCCGTA | CCTGAGCCTCCAGTGACTTC |
| **ChIP** | | |
| *Fosl2 (ChIP)* | CGGGCCCCAGTTATTTATTT | CCAGTGAGACATTCGGGAGT |
| *Tpm1*  *(ChIP)* | AAAACGCCTAGACCGCTACA | CTGCCCCTGATAGTGGACAT |

**Supplementary Table 3 shRNAs**

| **Target** | **Forward sequences (5’ → 3’)** | **Reverse sequences (5’ → 3’)** |
| --- | --- | --- |
| *Kdm4b shRNA* | CCGGTTCGGTGGACAGACGGTAATCCTCGAGGATTACCGTCTGTCCACCGAATTTTTG | AATTCAAAAATTCGGTGGACAGACGGTAATCCTCGAGGATTACCGTCTGTCCACCGAA |
| *Ccar1 shRNA1* | CCGGGCGCCGTTATCAGAACTTATACTCGAGTATAAGTTCTGATAACGGCGCTTTTTG | AATTCAAAAAGCGCCGTTATCAGAACTTATACTCGAGTATAAGTTCTGATAACGGCGC |
| *Ccar1 shRNA2* | CCGGCCTGTAATTTCTAGCATTCAACTCGAGTTGAATGCTAGAAATTACAGGTTTTTG | AATTCAAAAACCTGTAATTTCTAGCATTCAACTCGAGTTGAATGCTAGAAATTACAGG |
| *Ccar2 shRNA1* | CCGGGGTTCATCTCACTCCCTATACCTCGAGGTATAGGGAGTGAGATGAACCTTTTTG | AATTCAAAAAGGTTCATCTCACTCCCTATACCTCGAGGTATAGGGAGTGAGATGAACC |
| *Smarca4 shRNA1* | CCGGCGGCTCAAGAAGGAAGTTGAACTCGAGTTCAACTTCCTTCTTGAGCCGTTTTTG | AATTCAAAAACGGCTCAAGAAGGAAGTTGAACTCGAGTTCAACTTCCTTCTTGAGCCG |
| *Smarca4 shRNA2* | CCGGCGCCCGACACATTATTGAGAACTCGAGTTCTCAATAATGTGTCGGGCGTTTTTG | AATTCAAAAACGCCCGACACATTATTGAGAACTCGAGTTCTCAATAATGTGTCGGGCG |
| *Med1 shRNA1* | CCGGGTGTTACATCACGTCAGATATCTCGAGATATCTGACGTGATGTAACACTTTTTG | AATTCAAAAAGTGTTACATCACGTCAGATATCTCGAGATATCTGACGTGATGTAACAC |
| *Med1 shRNA* | CCGGTAAGCTTGTGCGTCAAGTAATCTCGAGATTACTTGACGCACAAGCTTATTTTTG | AATTCAAAAATAAGCTTGTGCGTCAAGTAATCTCGAGATTACTTGACGCACAAGCTTA |
| *P65 shRNA1* | CCGGAGGCCATATAGCCTTACTATCCTCGAGGATAGTAAGGCTATATGGCCTTTTTTG | AATTCAAAAAAGGCCATATAGCCTTACTATCCTCGAGGATAGTAAGGCTATATGGCCT |
| *P65 shRNA2* | CCGGCTGTCCTCTCACATCCGATTTCTCGAGAAATCGGATGTGAGAGGACAGTTTTTG | AATTCAAAAACTGTCCTCTCACATCCGATTTCTCGAGAAATCGGATGTGAGAGGACAG |

**Supplementary Methods**

**Reporter gene assay**

Reporter gene assays were performed as previously described ^1^. In brief, 293T cells were grown in 12-well plates and transfected with a pNF-κB-Luc plasmid and vectors for p65, KDM4B WT, KDM4B ΔFe, or CCAR1 for 36 h. Cells were lysed in Reporter Lysis buffer (Promega, E4030) and assayed for luciferase activity using SpectraMax i3x (Molecular Devices, CA, USA).

**Pharmacokinetic study of ML324**

In vivo pharmacokinetic study was carried out using ICR male mice (n=3). Animals were fasted for 12h before dosing and 2h after dosing. ML324 was prepared as a solution (DMSO:PEG400:DW=5:40:55%) and administered intravenously and intraperitoneally at a dose of 1.74 mg/kg. Blood samples were collected from orbital sinus under ether anesthesia at 5, 30, and 60 min and 2, 4, 8, and 24 h post dose. The plasma was separated from blood samples by centrifugation and stored at –20°C. The plasma concentration of ML324 was determined by the Agilent 1200 HPLC system interfaced to Agilent 6460A QQQ mass spectrophotometer (Agilent Technologies, CA, USA) controlled by Mass hunter software. The plasma concentration - time data were analyzed by non-compartmental analysis using WinNonlin version 4.1 (Pharsight, CA, USA).

**Immunohistochemistry**

The femur sections were blocked with blocking reagent (50 mM Tris-HCl, pH 7.5, 150 mM NaCl, 0.3% Triton X-100, and 5% normal goat serum) for 30 min at room temperature and incubated with anti-H3K9me3 antibody (Active motif, 39161, 1:250) at 4 °C overnight. Immunodetection was performed using ABC reagent (Vectorstain, PK-6100) and DAB (Vector Lab, SK-4100). Hematoxylin (Sigma, MHS1) was used for counterstaining ^2^.

**Supplementary References**

1 Lee, H. *et al.* Ethyl Acetate Fraction of Aqueous Extract of Lentinula edodes Inhibits Osteoclastogenesis by Suppressing NFATc1 Expression. *Int J Mol Sci* **21**, doi:10.3390/ijms21041347 (2020).

2 Kim, K. *et al.* VprBP has intrinsic kinase activity targeting histone H2A and represses gene transcription. *Mol Cell* **52**, 459-467, doi:10.1016/j.molcel.2013.09.017 (2013).
